# Age-related hearing loss and its potential drug candidates: a systematic review

**DOI:** 10.1186/s13020-023-00825-6

**Published:** 2023-09-20

**Authors:** Shiyu Hu, Qingru Sun, Fei Xu, Ninghua Jiang, Jianli Gao

**Affiliations:** 1 School of Pharmaceutical Sciences, Zhejiang Chinese Medical University, Hangzhou, 310053 Zhejiang People’s Republic of China; 2 School of Medical Technology and Information Engineering, Zhejiang Chinese Medical University, Hangzhou, 310053 Zhejiang People’s Republic of China; 3 Department of Pharmacy, The Second Affiliated Hospital of Jiaxing University, Jiaxing, 314000 Zhejiang People’s Republic of China

**Keywords:** Age-related hearing loss, Herbal medicine, Mitochondrial function, Anti-inflammation, Ion homeostasis, Ginsenoside Rb1

## Abstract

**Background:**

Age-related hearing loss (ARHL) is one of the main illnesses afflicting the aged population and has a significant negative impact on society, economy, and health. However, there is presently no appropriate therapeutic treatment of ARHL due to the absence of comprehensive trials.

**Objectives:**

The goal of this review is to systematically evaluate and analyze recent statistics on the pathologic classifications, risk factors, treatment strategies, and drug candidates of ARHL, including that from traditional Chinese medicine (TCM), to provide potential new approaches for preventing and treating ARHL.

**Methods:**

Literature related to ARHL was conducted in databases such as PubMed, WOS, China National Knowledge Infrastructure (CNKI), and Wanfang from the establishment of the database to Jan, 2023. The pathology, causal factor, pathophysiological mechanism, treatment strategy, and the drug candidate of ARHL were extracted and pooled for synthesis.

**Results:**

Many hypotheses about the etiology of ARHL are based on genetic and environmental elements. Most of the current research on the pathology of ARHL focuses on oxidative damage, mitochondrial dysfunction, inflammation, cochlear blood flow, ion homeostasis, etc. In TCM, herbs belonging to the kidney, lung, and liver meridians exhibit good hearing protection. Seven herbs belonging to the kidney meridian, 9 belonging to the lung meridian, and 4 belonging to the liver meridian were ultimately retrieved in this review, such as *Polygonum multiflorum* Thunb., *Panax ginseng* C.A. Mey, and *Pueraria lobata* (Willd.) Ohwi. Their active compounds, 2,3,4',5-Tetrahydroxystilbene-2-O-D-glucoside, ginsenoside Rb1, and puerarin, may act as the molecular substance for their anti-ARHL efficacy, and show anti-oxidative, neuroprotective, anti-inflammatory, anti-apoptotic, or mitochondrial protective effects.

**Conclusion:**

Anti-oxidants, modulators of mitochondrial function, anti-inflammation agents, vasodilators, K^+^ channel openers, Ca^2+^ channel blockers, JNK inhibitors, and nerve growth factors/neurotrophic factors all contribute to hearing protection, and herbs are an important source of potential anti-ARHL drugs.

## Background

**Fig. 1:**
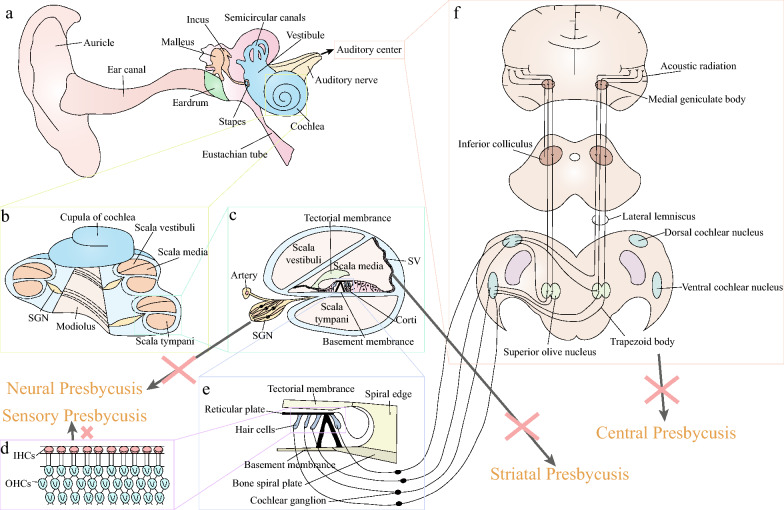
Inner ear anatomy and the pathological sites of different ARHL. **a** Anatomical whole structure of ear. The air conduction pathway of sound: sound wave → auricle → ear canal → eardrum → malleus → incus → stapes → vestibule → perilymph and endolymph → Corti → auditory nerve → auditory center. **b** Cochlear longitudinal profile. **c** Analysis of cochlear canal enlargement. **d** OHCs and IHCs. **e** Shear motion. **f** Auditory center

Human ear can detect sound waves from 20 to 20,000 Hz, with frequencies between 1,000 and 3,000 Hz are the most sensitive. There sound waves enter the inner ear by two different methods: air conduction (AC) and bone conduction (BC). BC allows sound waves to pass directly through the skull to excite the periosteum, and excite the Corti to produce auditory perception, only plays a role in auditory examinations. Contrarily, AC predominates in most cases (shown in Fig. 1a). In this way, sound waves are collected and transmitted by the external ear. After a series of treatments in the middle ear, it passes to the Corti in the inner ear to sense the sound, and finally to the auditory center to sense sound.

Sound wave vibrations cause vibrations in the basilar membrane of the cochlea, which then propagate forward as waves. Different locations of the cochlear basilar membrane correspond to sounds of each frequency: high-frequency sounds are amplified most in the cochlea's basal turn, which is close to the vestibular window, while low-frequency sounds are amplified most in the cochlea's apical turn, and mid-frequency sounds are amplified in the middle turn of the basilar membrane resonance. The basal turn of the basilar membrane of the cochlea vibrates only in response to high-frequency sounds, while low-frequency sounds cause most of the vibration of the basilar membrane.

Hearing loss is one of the most common conditions that interfere with a person's ability to work and lead a normal life. Auditory dysfunction and various degrees of hearing impairment are caused by qualitative or functional lesions of the auditory conduction pathways and collectively referred to as hearing loss. More than 1.5 billion individuals worldwide have some degree of hearing loss as of 2021, and this number may increase to 2.5 billion by 2050, according to estimates from the World Health Organization (WHO) [1].

Age-related hearing loss (ARHL), also known as presbycusis, is a condition in which hearing loss develops gradually over time in the majority of older people. It is the third most common disease in the United States after heart disease and arthritis among diseases that primarily affect older adults [2]. ARHL is described as progressive, bilateral, symmetrical, with most patients starting to lose hearing at high frequencies. It is also characterized by diminished auditory sensitivity and speech identification in noisy surroundings, and it causes communication impairments that progress to a range of mental illnesses such as social isolation, geriatric depression, cognitive obstacles, etc. [3]. Research has revealed a strong link between ARHL and Alzheimer's disease [4]. There were 14.566 million ARHL patients worldwide in 2019 [5]. As a result, ARHL also imposes a significant socioeconomic burden. The WHO reported that the economic burden caused by hearing loss is as high as US$1 trillion per year, and as people’s life expectancy increases, the economic cost of ARHL will continue to increase [1].

The pathophysiological causes and therapy options for ARHL are still poorly understood. As a result, there are no medications on the market right now that can effectively treat ARHL. However, there are many herbal medicines with great potential for treating ARHL. Traditional Chinese medicine (TCM) has a lengthy history of clinical application and a very rich body of knowledge regarding ARHL. In this review, we searched for literature in databases such as PubMed, WOS, China National Knowledge Infrastructure (CNKI), and Wanfang from the establishment of the database to Jan, 2023. The pathology, causal factors, pathophysiological mechanisms, treatment strategies, and the drug candidates of ARHL were extracted and pooled for synthesis. We summarize the types, causes, and pathophysiological mechanisms of ARHL, as well as therapy options for ARHL using both herbal remedies and contemporary medications, and their associated pharmacological consequences. This review provides a unique perspective on how herbal remedies and contemporary medications affect the mechanisms of ARHL, particularly in the treatment of ARHL with herbal remedies. We hope this will help develop drugs with potential therapeutic effects on ARHL.

## Types of ARHL and its pathology

ARHL mostly involves lesions of the inner ear, auditory nerve, and central nervous system, and is a type of sensorineural hearing loss. Based on the pathological circumstances of the inner ear of the human temporal bone after death and its audiogram study, ARHL can be classified into sensory presbycusis, striatal presbycusis or metabolic presbycusis, neurologic presbycusis, and cochlear conduction deafness [6]. The first three are collectively referred to as sensorineural hearing loss. However, the majority of ARHL patients exhibit a combination of multiple pathologies rather than just one. Lesions of the central auditory pathway may play a significant role in the development of ARHL in addition to those that affect the peripheral auditory system [7].

### Sensory presbycusis

In audiograms, sensory presbycusis is defined by an increase in the high-frequency threshold, and in temporal bone dissection, by the degeneration of hair cells (HCs) near the basal turn of the cochlea. HCs include outer hair cells (OHCs) and inner hair cells (IHCs) (Fig. 1d). Sensory presbycusis are susceptible to many factors, including external mechanical damage, noise exposure, ototoxic drug damage, etc. Bredberg analyzed sensory presbycusis in the cochlea of 125 people from newborns to over 90 years old and found that both the OHCs at the apical turn and basal turn of the cochlea degenerate with age [8]. Subsequent studies have confirmed that the cochlea sensory HCs degenerate from apical turn and basal turn of the cochlea toward the middle turn of the cochlea [9]. Since people with ARHL generally show an elevated high-frequency threshold, corresponding to the basal turn of the cochlea, while the loss of HCs in the low-frequency part has little effect on hearing thresholds, it is generally accepted that the basal turn of the cochlea HC loss is a major pathology of cochlear aging.

### Striatal presbycusis (metabolic presbycusis)

Striatal presbycusis, also known as metabolic presbycusis, manifests as atrophy of the stria vascularis (SV) in the temporal bone, flattening of the hearing curve on audiograms, or a minor increase in hearing threshold at high frequencies. SV cells are located on the outer wall of the cochlea duct (Fig. 1c) and are an important part of hearing in the cochlea. Cochlear morphology studies in quiet-grown gerbils suggest that the most important site of association with ARHL is SV [10]. SV cells can generate endocochlear potential (EP) [11, 12]. EP can help auditory receptors convert sound energy into neural impulses, which are then transmitted to the center of the auditory system to generate auditory sensations. EP is maintained by ion fluxes in the SV and spiral ligament (SL) located on the lateral cochlear wall [12, 13]. It may be argued that the atrophy and malfunction of the SV is a significant contributor to ARHL, since the loss of EP plays a significant role in the rise of the hearing threshold in mammals.

### Neural presbycusis

Neural presbycusis is characterized by degeneration of the auditory nerve and loss of cochlear neurons throughout the cochlea. The degeneration of spiral ganglion neurons (SGNs) may be caused by the accumulation of noise-induced loss of cochlear-nerve/hair-cell synapses [14, 15]. The cochlea's apical and basal turns are where nerve damage occurs most frequently in both humans and animals [16, 17]. Low spontaneous firing rate nerve fibers are the most vulnerable parts of the SGN and suffer more severe damage than medium and high spontaneous firing rate fibers [18]. The spontaneous firing rate of the auditory afferent nerve is negatively correlated with its threshold, that is, fibers with a high spontaneous firing rate have the lowest response threshold, while fibers with a low spontaneous firing rate have the highest response threshold. Thus, loss of nerves with low spontaneous firing rate fibers results in loss of high-frequency hearing. In mammals, neuronal loss is more severe than sensory HCs loss, according to a number of studies [9, 19]. This finding raises the possibility that neuronal loss is not merely a secondary process to the loss of sensory HCs, and that the degree of neuronal damage may actually be greater.

### Central presbycusis

Unlike other sensory systems, which generally have only one or two centers, the auditory system has four centers: cochlear nucleus, superior olive nucleus, lateral lemniscus, and inferior colliculus (Fig. 1f). The cochlear nucleus receives sound information from the HCs via the SGNs that has been processed by the peripheral auditory system [20]. Each level of the central nerve is divided into various sections according to its anatomical position, and each section is further divided into numerous smaller sections according to the type of neuron it contains. Therefore, the central pathway of the auditory system is very complex, and the physiological and pathological mechanisms of the auditory system center are still unclear, and there is no reference standard for age-related central auditory disorders [21]. It has been suggested that ARHL is associated with decreased volume in the right temporal lobe [22]. The structural MRI study found that ARHL was independently related to the accelerated atrophy of total brain size and regional brain size and the decrease of white matter integrity [23]. Findings of synaptopathy in the auditory nerve center suggest that synapses degenerate during aging, and more so in non-type I_a_ auditory nerve synapses, and may therefore play an important role in the development of ARHL [24].

## Major causal factors of ARHL

### Biological aging

Hearing loss can begin in adolescence, but most hearing loss due to biological aging begins much later, always at age 60. Numerous studies have demonstrated that as animals get older, their ABR threshold rises [25]. Biological aging induced hearing loss is usually caused by oxidative damage accumulated during aging and gradual loss of HCs [26, 27]. It is initially more pronounced at high-frequencies and then spreads to mid-frequency and low-frequencies. Aged dogs and mice were similarly found to have significantly less spiral ganglion neurons, inner and outer HCs at the basal turn of the cochlea [28, 29]. In the aging Long Evans and Fischer 344 rats, the number of neurons containing non-phosphorylated neurofilament and their protein level in the central auditory system decreased significantly, indicating that aging has a very important contribution to the central auditory system [30]. These findings suggest that biological aging plays a critical role in the development of ARHL.

### Genetics

Because congenital deafness and early-onset deafness are mostly related to genetic factors [31], ARHL is expected to be affected by genetic factors. There have been 124 non-syndromic hearing loss genes discovered to date [32], including 77 autosomal recessive nonsyndromic hearing loss genes, 51 autosomal dominant nonsyndromic hearing loss genes (11 genes GJB2, TMC1, MYO7A, TECTA, MYO3A, MYO6, COL11A2, PTPRQ, TBC1D24, CEACAM16, RIPOR2/FAM65B are both autosomal recessive genes and autosomal dominant genes). In addition, there are 5 X-linked genes, a small number of which are inherited through maternal mitochondria and one, DFNY1, is inherited through the Y chromosome. In addition, there are 11 different types of syndromes of hearing loss, including Alport Syndrome and Branchio-Oto-Renal Syndrome. Numerous researchers are presently attempting to pinpoint the genes responsible for ARHL, but no credible candidates have emerged. A genome-wide association study (GWAS) of 100 inbred mouse strains identified several statistically significant loci that may predispose animals to hearing impairment caused by noise exposure [33]. These genes may also contribute to ARHL in humans. Numerous genes that may contribute to ARHL were found in a GWAS study of European populations, but none of them stood out statistically above the rest [34]. This impact may be brought about by a number of circumstances. First, a large number of genes work together to cause ARHL, not just a single gene. Second, ARHL is caused by a variety of environmental/external factors in addition to genetic changes. The genes responsible for ARHL mainly include deafness-causing single genes, neurotransmitter-related genes, oxidative stress-related genes, and mitochondrial function-related genes. The following is a description of the few ARHL-related genes that are now the focus of intensive research, which are shown in Table 1.

**Table 1:** Genes associated with ARHL

Genetics	Types	Related mechanisms	Refs.
GRHL2	Deafness-causing single gene	The protein encoded by this gene is a transcription factor associated with deafness; associated with ARHL in European populations	[35]
Slc4a10		Mutant mice have elevated ABR thresholds; cochlear OHC loss is evident	[36]
SLC9A3R1		Hearing impairment in mutant zebrafish larvae	[39]
KCNQ4		May have effects on DFNA5	[40]
Ahl		Deletion of this gene results in disordered cochlear HCs, raising the ABR threshold	[42, 43]
GJB2		This gene encodes Cx26, and deficiency of Cx26 impairs active cochlear amplification	[44]
A430005L14Rik		Homozygous mutant mice exhibit asynchrony in auditory nerve firing during noise	[45]
Dclk1		Homozygous mutant mice had significantly higher hearing thresholds	[45]
GRM7	Neurotransmitter-related genes	The gene is able to increase the release of glutamate, bringing it to a toxic state that increases the risk of ARHL	[49]
NAT2	Oxidative stress-related genes	This gene is involved in the metabolism and detoxification of ROS, and the polymorphism of NAT2*6A is associated with ARHL in the population	[51]
GSTT1		GSTT1 null genotype was significantly associated with ARHL	[52]
SOD2 promoter variant (−38C > G)		May be associated with male ARHL	[53]
UCP2Ala55Val		UCP2 is able to control ROS, and the UCP2Ala55Val polymorphism was significantly associated with ARHL in the Japanese population	[55]
mtDNA 4977-bp	Mitochondrial function-related genes	Deletion of this gene may be associated with ARHL	[56]
mtDNA haplogroup A		Associated with increased hearing loss in Japanese men	[58]
mtDNA haplogroup N9		Associated with decreased hearing loss in Japanese women	[58]

#### Deafness-causing single gene

The granular head-like 2 gene (GRHL2) is located on chromosome 8, and the protein it encodes is a transcription factor that is associated with Deafness, Autosomal Dominant 28 (DFNA28), which is associated with ARHL in Europeans [35], but not in Han Chinese [36], which may be due to population differences. Through the N-ethyl-N-nitrosourea (ENU) mutagenesis protocol in mice, it was reported that Slc4a10, encoding solute carrier family 4, sodium bicarbonate transporter, member 10, is a novel late-onset hearing loss gene, critical to inner ear homeostasis [37]. Animal studies have supported the link between this gene and hearing loss [38]. Two unrelated Italian men with ARHL were found to have the heterozygous missense mutation c.539G > A, p. (R180Q) in the SLC9A3R1 gene, which alters the PDZ2 domain of the NHERF1 protein [39]. The homozygous zebrafish larvae with this gene mutation have obvious hearing loss, but no damage to HCs and neurons was observed [39]. Mutations in the potassium voltage-gated channel subfamily q member 4 (KCNQ4) gene have a large contribution to DFNA5 [40], it has also been shown that there is no significant link between DFNA5 and ARHL [41]. Ahl (age-related hearing loss (AHL) gene) is one of the main reasons for ARHL susceptibility. Ahl1 is located on chromosome 10 [42, 43]. The deletion of the Ahl gene causes disorders in the IHC and OHC of the cochlea, which increases the animals’ hearing threshold. Connexin 26 (Cx26) and connexin 30 (Cx30), encoded by GJB2 and GJB6, respectively, are the major protein subunits in the cochlear gap junction. Mutations in GJB2 are the most common genetic cause of loss of active cochlear amplification [44]. Furthermore, mice with homozygous mutations in the A430005L14Rik and Dclk1 genes experience hearing loss [45]. The above studies suggest that there are many single genes that cause deafness, but very few that are directly associated with ARHL, mainly Ahl.

#### Neurotransmitter-related genes

The strongest candidate for the ARHL gene is GRM7, a gene that encodes the glutamate metabolic receptor type 7. Glutamate is an excitatory neurotransmitter, impairment of glutamate homeostasis has important neuropathological consequences and is associated with a variety of neurological or neurodegenerative diseases [46]. The presence of excessive glutamate is believed to mediate the auditory neuronal neurotoxicity [47]. And GRM7 is considered to be the core for maintaining glutamate synaptic transmission and the steady state of the processes between HCs and afferent auditory nerve fiber dendrites in the mammalian cochlea [48]. The pathogenic allele of GRM7 changes the synaptic autoregulation of glutamate in the synaptic fissure of the sensory cells of the IHC and auditory neurons, leading to an increase in the level of glutamate in the fissure, followed by the death of excitotoxic neurons and/or sensory cells [49]. Therefore, the GRM7 mutation may lead to the ARHL vulnerability. A study that analyzed hearing-related genes in the hippocampus of a BXD mouse strain also confirmed that impaired glutamatergic synaptic pathways are involved in ARHL [50].

The above suggests that the neural-related genes responsible for ARHL are mostly associated with the secretion of the excitatory neurotransmitter glutamate. Excessive secretion of glutamate can cause severe damage to neurons and HCs, leading to ARHL.

#### Oxidative stress-related genes

N-acetyltransferase 2 (NAT2) is involved in the metabolism and detoxification of reactive oxygen species (ROS). The DNA of 68 patients with ARHL and 98 healthy controls was studied to prove that the polymorphism of NAT2*6A was significantly associated with ARHL in the population [51]. Glutathione S-transferase (GST) is an anti-oxidant enzyme, GSTM1 and GSTT1 null genotypes are also significantly associated with ARHL [52]. A second antioxidant enzyme, mitochondrial superoxide dismutase 2 (SOD2), has a promoter variation (−38C > G) that has been linked to an increased risk of ARHL in males [53]. The main function of mitochondrial uncoupling protein 2 (UCP2) is to control ROS [54]. A polymorphism of UCP2 Ala55Val has been reported to be significantly associated with ARHL in the Japanese population [55].

Oxidative stress is an important trigger for biological aging, and its impact on ARHL is also significant. Therefore, interfering with the oxidative stress-related genes that cause ARHL is also a treatment for ARHL.

#### Mitochondrial function-related genes

Mitochondrial DNA (mtDNA) 4977-bp deletions are often found in ARHL patients. In 17 ARHL patients and 17 normal subjects, 14 patients and 8 normal subjects showed 4,799-bp deletion respectively [56]. Five ARHL patients' samples were examined, and it was discovered that many mitochondrial mutations in the peripheral auditory system [57]. Thus, acquired mtDNA mutations are one of the determinants of ARHL. Analysis of 12 major mtDNA haplotype groups (D4a, D4b, D5, G1, G2, M7a, M7b, A, B4, B5, N9 and F), and found the risk of hearing loss was significantly increased in Japanese male subjects belonging to haplogroup A, whereas the risk of hearing loss was significantly decreased in Japanese female subjects belonging to haplogroup N9, suggesting that mitochondrial haplogroups may be associated with ARHL [58].

The above studies suggest that interference with mtDNA mutations in the auditory system is also a potential treatment for ARHL.

### Living environment and life-style

In recent years, patients with ARHL have tended to be younger; one reason for this is prolonged exposure to noise. The degeneration of HCs of basal turn of the cochlea, especially the massive loss of OHCs, may be triggered by noise exposure [59]. In old age, animals bred in quiet environments lost much less HCs [60]. In one study, noise exposure in young (2-month-old C57BL/6J mice) significantly sped up or aggravated the course of ARHL [61]. Workers exposed to noise have a markedly increased risk of developing hearing loss and tinnitus compared to those who operate in quiet workplaces [62]. Noise-induced hearing loss (NIHL) and ARHL may be interrelated, as young people of the Mabaan tribe living in the Sudanese desert have a similar level of hearing to young people in other regions, but they have better hearing in old age than in other regions, presumably as a result of their peaceful environment and healthy lifestyle [63]. Another survey shows that compared to natives living on Easter Island, men who are living or have lived in Chile have significantly lower hearing than men who always lived on Easter Island [64]. In animal models, there was also found degeneration of the SL, which correlates with SV degeneration, in the cochlea of quiet-aged gerbil [65]. These studies all suggest that noise is an important factor in accelerating the development of ARHL in young people.

Lifestyle may affect auditory performance and progression of ARHL at any age. Smokers or passive smokers are also at high risk of developing ARHL, with passive smokers having a higher risk, and moderate alcohol consumption can reduce the risk of ARHL [66]. A survey of the Korean population showed that smokers with diabetes had a higher risk of developing ARHL than non-smokers with diabetes [67]. In the Malaysian population, an increased risk of ARHL was linked to an increased waist circumference, decreased niacin and potassium intake, and increased carbohydrate intake [68]. Intermittent hypoxic conditions, high-fat diet, and galactose injections accelerate the aging of cochlear HCs in mice [69]. High body mass index (BMI) was also significantly associated with ARHL [70]. Higher intakes of sugary foods, high-calorie beverages, and beer were associated with an increased risk of ARHL [71], but higher intakes of seeds and nuts, fruits, and seaweed, and higher intakes of riboflavin, niacin, and retinol could reduce the incidence of ARHL in the Korean population [72, 73]. In Korean adult ARHL patients, the incidence rate of moderate to severe (but not mild) ARHL is significantly positively correlated with sleep duration [74]. Additionally, other conditions like diabetes, hypertension, elevated serum cholesterol, and other conditions have a strong link to ARHL [75]. These unhealthy lifestyle habits and hearing loss caused by chronic diseases are often associated with oxidative stress [76].

Therefore, people can prevent the occurrence of ARHL by maintaining good lifestyle habits such as limiting the intake of high-calorie foods, quitting smoking, moderate alcohol consumption, moderate exercise, and maintaining good sleep.

### Ototoxic drugs

Another reason why ARHL patients tend to be younger is the use of ototoxic drugs. Exposure to ototoxic drugs, such as aminoglycoside antibiotics (such as streptomycin, gentamicin, acamicin, etc.), non-steroidal anti-inflammatory drugs, loop diuretics, antineoplastic drugs (such as cisplatin, nitrogen mustard, etc.), or inhalation of harmful gases (carbon monoxide, hydrogen sulfide, etc.), all can cause ARHL. Cisplatin is an anticancer drug that has shown clinical efficacy against a variety of cancers. Tinnitus and high-frequency hearing loss are side effects of cisplatin use [77, 78]. The ototoxicity of cisplatin occurs in 23–50% of adults and up to 60% of children. Some studies have reported that up to 100% of cancer patients treated with cisplatin have an increased hearing threshold [79]. The ototoxicity of aminoglycoside drugs is associated with a higher concentration of the drug in the internal lymph fluid, which impairs the energy production and utilization of the OHCs and causes dysfunction of Na^+^-K^+^-ATPase on the cell membrane, resulting in damage to HCs. Early changes caused by aminoglycosides are reversible, but beyond a certain point they become irreversible damage, with a 63% chance of hearing loss in people using aminoglycosides [79, 80].

Noise exposure and the use of ototoxic drugs can independently contribute to hearing loss in individuals of any age, but they are also important external factors that exacerbate the progression of ARHL. There are many interesting similarities between age-related, noise induced, and drug-induced hearing loss, such as the potential involvement of ROS and apoptosis and necrotic cell death [81]. Therefore, the treatment methods for hearing loss due to noise exposure and ototoxic drugs may also be cited in ARHL. In everyday life, the use of ototoxic drugs and exposure to noise should be avoided to avoid the occurrence of ARHL, but this also suggests that the development of new cancer treatment drugs with fewer side effects is also urgent.

### Inflammation and autoimmunity

Many patients had a history of colds before the onset of ARHL, and the serological examination of the virus also demonstrated that many of the viruses were associated with hearing loss, such as Ménière's disease, and vestibular neuritis/viral labyrinthitis [82]. The inner ear is considered immune free because the presence of a blood-labyrinth barrier restricts the entry of blood cells into the inner ear [83]. However, some studies have shown that cochlear immune cells exist in the spiral ganglion, spiral ligament and stria vascularis [84]. In the cochlea, noise exposure stimulates the capillaries—perivascular units of the SV and concentrates circulating immune cells in the cochlea [85], which in turn affects ion circulation and EP, ultimately causing ARHL. The aging cochlea exhibits high concentrations of cytokines like tumor necrosis factor (TNF) and interleukin 1β (IL-1β) [86]. In addition, studies have found a decrease in the expression of T follicular helper cells and B cells in the ARHL group [87]. Therefore, studying inflammation in order to investigate treatment options for ARHL is also a potential approach.

### Others

Women typically have better high-frequency hearing sensitivity, while males typically have better hearing at low frequencies than women [88], and men have much more severe ARHL than women of the same age [71, 72]. It may be related to estrogen, which has anti-oxidative properties and neuroprotective effects. Older women with ARHL treated with estrogen have better recovery than older women with ARHL not treated with estrogen [89], and women with Turner syndrome lack estrogen, they have a higher risk of sensorineural hearing loss than normal women [90]. ARHL prevalence is much lower in non-Hispanic blacks than in Hispanic whites [91], which may be related to differences in melanin levels in the body. Therefore, estrogen and melanin may also be potential therapeutic drugs for ARHL.

## Pathophysiological mechanisms and treatment strategies of ARHL

### Oxidative damage—anti-oxidants

It is widely believed that oxidative damage is an important cause of ARHL. In energy metabolism, cells are able to continuously reduce oxygen through the respiratory chain to superoxide and finally to ROS. NADPH oxidase (NOX) is an important source of ROS in the cochlea, and NOX activity increases the expression of genes involved in the excitatory pathway, damages the cochlea, and ultimately causes ARHL [92]. However, cells can produce free radical scavengers or anti-oxidant enzymes such as SOD, catalase (CAT), glutathione peroxidase (GPx), glutathione reductase (GR), etc. to inhibit ROS [93]. ROS can also be inhibited by supplementing non-enzymatic anti-oxidants, such as vitamin C, vitamin E, glutathione (GSH), flavonoids, etc. [94]. Under normal circumstances, a balance is maintained between ROS and anti-oxidant enzymes, which regulate various physiological processes, such as regulating signal transduction, inducing cell mitosis, etc. When this equilibrium is upset, an excess of ROS is produced, which can lead to oxidative stress. There are many factors that can cause oxidative damage, such as aging, mechanical damage, and inflammation. Oxidative damage is strongly associated with neurodegenerative diseases, including ARHL. Studies have shown that exposure to H_2_O_2_ causes mouse auditory HCs to prematurely exhibit a senescence-like phenotype [95]. And mice lacking SOD1 exhibited reduced HCs, atrophy of SV, and degeneration of SGNs [96]. As a result, many studies have been conducted to prevent or treat ARHL by developing anti-oxidants.

Anti-oxidants, including vitamin C, vitamin E, GSH, coenzyme Q (CoQ), carotenoids, and melatonin are frequently utilized to combat oxidative damage [94, 97]. Melatonin has strong anti-oxidant capacity that helps prevent or delay the dysfunction of cochlear OHCs [98], as well as the ototoxicity caused by cisplatin [99] and noise exposure-induced hearing loss [100]. However, melatonin cannot completely neutralize the occurrence of ARHL [101]. α-lipoic acid is a good anti-oxidant that has been shown to successfully prevent ARHL in rats [102]. A protective effect of vitamin C intake on ARHL in older adults has also been observed [103]. Additionally, N-acetylcysteine (NAC, an antioxidant and precursor to GSH) exhibits superior hearing protection to vitamin A by supplying cysteine for the production of GSH, which prevents oxidative damage to the rat auditory system [104]. However, another study showed that while a diet rich in anti-oxidants such as vitamins A, C, and E, L-carnitine, and α-lipoic acid significantly increased the anti-oxidant capacity of inner ear tissue in CBA/J mice, it did not delay the progression of ARHL [105]. Moreover, atorvastatin may protect hearing by modulating the HSF1/Sirt1 pathway to suppress oxidative stress and reduce HCs death and SGN mitochondrial damage in the cochlea of elderly CBA/J mice. However, the fixed dose combination with 5-HT2A receiver antiagonist, SAP, cannot prevent ARHL [106].

### Mitochondrial dysfunction—modulators of mitochondrial function and metabolism

Mitochondrial dysfunction in cochlear cells can be caused by inhereditary mtDNA mutations, accumulation of acquired mtDNA mutations with age, mitochondrial overdrive and calcium dysregulatio, or accumulation of ototoxic drugs in hair cell mitochondria [107]. The Sirtuin family is a nicotinamide adenine dinucleotide (NAD^+^)-dependent histone deacetylase with seven members (SIRT1-7), is involved in many crucial biological processes, including apoptosis, endocrine signaling, glucose homeostasis, aging, and longevity [108]. It is also capable of regulating the circadian clock and mitochondrial biogenesis [109].

Resveratrol, a natural polyphenolic compound that could activate SIRT1, is found in a variety of plants, such as grapes, berries, mulberries and peanuts [110]. Resveratrol has a good protective effect on cisplatin [111], gentamicin [112] and noise-induced [113] hearing loss. Studies have shown that early treatment with resveratrol can significantly reduce hearing thresholds at all frequencies, and can reduce the mRNA expression levels of pro-apoptotic genes Bax and Bak, and increase the mRNA expression level of anti-apoptotic genes Bcl-2 and Bcl-xL, but there was no significant difference in hearing levels between the late treatment group and the control group [114]. However, it is worth noting that high doses of resveratrol will cause more serious loss of OHCs and IHCs in elderly mice [115]. Overexpression of miR-34a would inhibit SIRT1, and resveratrol treatment was shown to significantly reduce miR-34a overexpression-induced HCs death in C57BL/6 mice [116]. Additionally, NRH, the reduced form of nicotinamide ribose (NR), ameliorated aminoglycoside-induced hearing loss and attenuated HC damage by increasing NAD^+^ levels and activating SIRT1 in the cochlea [117].

### Inflammation and autoimmunity—anti-inflammation

A variety of factors can cause cochlear inflammation, such as biological aging, noise, otitis media, use of ototoxic drugs, radiation damage, etc. After the cochlea is damaged, TNF-α is expressed in the cochlea, which in turn stimulates other cytokines such as IL-1β and IL-6 to concentrate in the cochlea, thereby increasing the production of free radicals in the auditory system and causing hearing loss [118, 119]. Therefore, the development of anti-inflammatory agents for the treatment of ARHL is currently a more general approach.

Currently, the most widely used medication for sudden deafness is corticosteroid, an anti-inflammatory drug. These drugs are usually taken orally or injected into the body (called systemic corticosteroid), but they can also be directly injected into the middle ear through the tympanic membrane (called corticosteroid in the tympanic cavity) [120]. The effect of glucocorticoid on auditory function was first reported in the late 1970s, when two synthetic analogues of glucocorticoid (cyclophosphamide and dexamethasone) were used in patients with autoimmune hearing loss, and hearing was substantially improved [121]. Glucocorticoids directly upregulate the levels of anti-inflammatory cytokines (such as IL-10) and downregulate pro-inflammatory cytokines (such as IL-1, IL-6, and TNF-α) to downregulate the inflammatory response [122]. However, steroid therapy is associated with many adverse reactions, including but not limited to hypertension, hyperglycemia, gastrointestinal problems, and dizziness. Blocking IL-1, IL-6 and TNF-α has been shown to be effective as a treatment in animal models. Anti-IL-6 receptor antibodies, such as MR16-1, offer functional and pathological protection in noise-damaged cochleas, mostly by lowering the inflammatory response [123]. Etanercept can inhibit TNF-α and improve the cochlear microcirculation [124]. Anakinra, an IL-1 receptor antagonist, can bind to the IL-1 receptor and prevent IL-1α and IL-1β activity [125]. Another study inoculated CD4^+^ T cells lacking regulatory T cells and IL-1 receptor type 2-expression T cells into a mouse model of ARHL and found that it prevented ARHL development and SGN degeneration [126], which may serve as a novel approach to the treatment of ARHL.

### Reduced cochlear blood flow—vasodilators

The labyrinthine arteries, which are virtually the only blood supply arteries to the inner ear, provide the majority of the blood flow to the inner ear. The inner ear's ability to function is significantly impacted by its lesions. According to some animal studies, EP disappears after 6 s of ischemia in the inner ear. Even when the blood supply is restored after 30 min of ischemia, the loss of EP remains irreversible. Therefore, cochlear microcirculation abnormalities are one of the causes of NIHL [127], ARHL [128], and ototoxic drug-induced hearing loss [129].

Several vasodilators have been shown to have the potential to treat different types of hearing loss by improving microcirculation in the blood-labyrinth barrier, maintaining EP, maintaining cochlear endolymphatic production, and reducing the build-up of toxic and inflammatory substances in the cochlea. The combination of cochlear vasodilator (Mg^2+^) and antioxidant free radical scavenger can better improve hearing function [130]. Calcium channel antagonists such as nimodipine and flunarizine hydrochloride capsules, for example, dilate blood vessels and relieve vasospasm, improve blood supply and lymph circulation in the inner ear, and inhibit the inflow of extracellular Ca^2+^. They also reduce the concentration of intracellular Ca^2+^. Alternatively, histamine injections can be used as an adjuvant to increase the permeability of the inner ear to dexamethasone. Intratympanic injection of 1% histamine and steroids may be a safe and effective treatment for hearing loss [131]. In addition, histamine also has a strong vasodilation effect, which increases the permeability of capillaries and small veins, and may improve the microcirculation of blood in the inner ear, promoting blood flow in the inner ear and thus potentially having a positive effect on the ARHL treatment.

### Disruption of ion homeostasis—K^+^ channel openers, Ca^2+^ channel blockers

The cochlea is spiral-shaped and consists of scala vestibuli, scala media and scala tympani (Fig. 1c), the scala media is the fluid zone with high K^+^ and low Na^+^ concentrations, and its unique extracellular solution called endolymph, while the scala vestibuli and scala tympani are low K^+^ and high Na^+^ area, which surround the scala media, constitutes the cochlear perilymph. As seen in Fig. 1e, the basilar membrane shifts after the sound wave reaches the cochlear perilymph. The basilar membrane and the tectorial membrane attached to the spiral limbus move up and down along different axes, causing the staggered movement between the tectorial membrane and the cuticular plate. This motion is a shear motion, which in turn bends or deflects the apical stereocilia of HCs located within the endolymph. The K^+^ channel at the top of the HCs opens at this point, allowing K^+^ from the endolymph to enter the cell and cause depolarization. This in turn opens the intracellular Ca^2+^ channel, allowing Ca^2+^ to enter the cell, which stimulates the release of neurotransmitters from the HCs, which then stimulates the nerve endings to produce nerve impulses that travel through the central pathway to the auditory cortex and result in hearing. Na^+^-K^+^-2Cl^−^ co-transporter isoform 1 (NKCC1) is expressed in SV, which can regulate the concentration of K^+^ in the scala media and play a key role in the production of EP. Studies have found that the expression of NKCC1 has declined with the development of ARHL [132]. Na^+^-K^+^-ATPase circulates K^+^ from the perilymph to the endolymph to regulate the resting potential in the scala media, and studies have found that the decrease of EP amplitude was significantly related to the stria vascularis and spiral ligament fibrocytes (type II), which are rich in Na^+^-K^+^-ATPase [133]. Cav1.3 is a voltage-gated calcium channel. In the cochlea of C57BL/6J mice, Cav1.3 expression rapidly reduced with age [134]. Hearing impairment and higher cochlear HCs loss were seen in aged C57BL/6J male mice after Cav1.3 was knocked down [135]. As a result, several studies have developed drugs that interfere with ion channels to prevent or treat ARHL.

K^+^ channel openers, such as zinc pyrithione, retigabine, and Maxipost, can restore K^+^ channel function and prevent hearing loss and tinnitus caused by aminoglycosides and salicylates [136, 137]. Numerous Ca^2+^ channel blockers have also demonstrated protective effects against ARHL. For instance, T-type Ca^2+^ channel blockers were able to significantly preserve SGN [138, 139] while also affecting the function and morphology of OHCs in C57BL/6J mice [140].

### Sensorineural death or damage—JNK inhibitor and nerve growth factor/neurotrophic factor

Damage to the inner ear causes the death of HCs, with OHCs usually being lost first, followed by IHCs and finally the supporting cells of the organ of Corti. In general, apoptosis is controlled by pro-apoptotic factors (caspases family and cytochrome c (Cyt-c)) and anti-apoptotic factors (Bcl-2 family) [141, 142]. Here, several mediators can regulate apoptosis of HCs, including c-Jun N-terminal kinase (JNK), which activates apoptosis by upregulating the transcription of the pro-apoptotic gene such as TNF-α, Fas-L, and Bak [143]. Activation of JNK/MAPK signaling and Bax in OHCs after exposure to intense noise, ototoxic drugs and aging has been shown to induce hearing loss [144–146]. D-JNKI-1, a cell-penetrating peptide that blocks JNK signaling, has been shown to be protective against aminoglycoside- and NIHL-induced hearing loss and against HCs loss [147]. B581 (50 microM) and FTI-277 (10 microM), which inhibit RAS, an upstream activator of the JNK pathway, were likewise effective at preventing gentamicin-induced HCs damage [148].

As previously mentioned, cochlear neuron loss plays a significant role in ARHL. Nerve growth factor (NGF) levels in the blood were significantly lower in patients with sensorineural hearing loss [149]. Studies have demonstrated that insulin-like growth factor 1 (IGF-1) deficiency and susceptibility to NIHL are directly related because Igf1^±^ mice had worse hearing progression after noise stimulation and lower plasma levels of IGF-1 [150].

Several growth factors prevent degeneration of cochlear neurons, such as NGF [151], neurotrophic factor-3 (NT-3) [152], which have been shown to prevent ototoxic drug-induced hearing loss, NIHL and ARHL. However, some studies have found that the presence of hypertension and delayed treatment are negative factors related to the effectiveness of NGF treatment [153]. IGF-1 has also been shown to protect cochlear HCs by activating the PI3K/Akt and MEK/ERK pathways, thereby improving hearing [154]. Typical nutritional neuromodulators, such as vitamin B12, can also nourish the nerves and restore nerve vitality in the inner ear.

### Mechanical assisted therapy—hearing aids, cochlear implants

A hearing aid is a device that amplifies sound to improve the perception of sound by a hearing loss patient. Hearing aids do not restore a patient’s damaged hearing, but they can use the patient’s residual hearing to enhance the patient’s speech recognition ability and improve the patient’s quality of life and work. It is currently the most widely used treatment for hearing impaired patients. Following clinical hearing aid therapy, the ARHL group showed a reversal of cross-modal reorganization of visual to auditory cortex, which was accompanied by alterations in speech perception and cognitive function [155].

A cochlear implant is an electronic device that reconstructs or acquires hearing for patients with severe, profound or total hearing loss. It mimics the function of the cochlea and converts acoustic signals into electrical signals, replaceing the function of damaged HCs and directly stimulating the SGN, which then transmits the electrical signals to the brain to produce hearing. Cochlear implants are an option to help if a person has significant hearing loss and hearing aids are insufficient to enhance sound in that situation. Through the cognitive function test of the elderly one year after the cochlear implant operation, it was found that the cognitive benefits of the elderly with cognitive impairment before the cochlear implant were even greater than those of the subjects with normal cognition [156].

However, cochlear implants require surgery, which many individuals decide against because of concerns about getting older and the state of the economy. And cochlear implant surgery can induce cellular inflammatory reaction and foreign body reaction, leading to cochlear injury, which may further aggravate hearing loss [157]. Moreover, the resolution and processing ability of the auditory system of hearing aids and cochlear implant are still insufficient, which limits the patient's perception of some subtle sounds [158]. And some patients have difficulty adapting to amplified sounds, resulting in many not using hearing aids [159].

### Others

Hyperbaric oxygen therapy: A controlled trial of 60 ears with hearing impairment and tinnitus that received hyperbaric oxygen therapy and 60 ears that received normoxic therapy found that patients treated with hyperbaric oxygen therapy had significantly better hearing and tinnitus recovery [160]. Recent studies have found that the combination of alprostadil and hyperbaric oxygen can significantly promote hearing recovery in patients with sudden sensorineural hearing loss by reducing blood viscosity and improving coagulation function [161]. Hyperbaric oxygen treatment may therefore be a useful complementary therapy. Otherwise, transplanting stem cells and embryonic neurons into the inner ear is also a strategy at the experimental research stage.

## Herbal medicines for hearing loss

Most patients with ARHL or sensorineural hearing loss have no specific cause of hearing loss, and systemic or intratympanic steroid therapy has become a common treatment for patients with sensorineural hearing loss [162]. However, because steroid therapy is associated with a number of adverse effects, such as, but not limited to, high blood pressure, high blood sugar, gastrointestinal issues, and dizziness, patients with these conditions cannot be treated with steroids, and these conditions, as well as ARHL, are widespread in older adults. Herbal medicines have a number of benefits, including fewer side effects, multiple targets, and a long history, making them increasingly popular as a therapeutic option worldwide. Many herbal medicines have shown promising efficacy in ARHL, which is of important for future drug development in ARHL. The following is a detailed overview of several herbal medicines related to hearing loss research and their possible active ingredients. According to the theory of TCM and herbal medicine theory, these medicines can be divided into kidney, lung and liver meridian. Table 2 summarizes the herbal medicines with hearing protection and their active ingredients and pharmacological effects.

**Table 2:** Herbal medicines with hearing protection and their active ingredients and pharmacological effects

Meridian	Herbal medicines	Main active compound	Monomer structure	Main pharmacological action	Target cell or target protein or target organ	Refs.
Drugs related to kidney meridian in herbal medicines	*Polygonum multiflorum* Thunb	2,3,4’,5-Tetrahydroxystilbene -2-O-β-D-glucoside		(1) Anti-oxidant; (2) Anti-apoptotic; (3) Anti-autophagy	(1) Nrf2, HO-1, NQO1; (2) Sesn2 / AMPK / mTOR	[166, 167]
	*Drynaria fortunei* (Kunze) J.Sm	Flavanoid component		HCs protection	IHCs and OHCs	[170]
	*Cornus officinalis* Sieb.et Zucc	Ursolic acid		Anti-oxidant	CAT and GPx	[173]
	*Rehmannia glutinosa* Libosch	Ethanolic extract of the steamed root of *Rehmannia glutinosa* Libosch	/	Anti-oxidant	anti-oxidant enzymes	[175, 176]
	*Sesamum indicum* L	*Sesamum indicum* L. oil	/	HCs protection	(1) Tecta gene; (2) HCs	[177]
		Sesamin				
	*Tripterygium wilfordii* Hook. f	Celastrol		(1) HCs protection; (2) Anti-oxidant	(1) Nrf-2, JNK, HSP32 / HO-1 (2) IHCs and OHCs; (3) Inner ear stem cells; (4) Atoh 1 gene	[178–180]
	*Poria cocos* (Schw.) Wolf	/	/	/	/	[181]
Drugs related to lung meridian in herbal medicines	*Pueraria lobata* (Willd.) Ohwi	Puerarin		Neuroprotection	(1) PKCγ; (2) GABABR1 and GABABR2	[185]
	*Astragalus membranaceus* (Fisch.) Bge	Astragaloside IV		(1) SV protection; (2) Anti-oxidant; (3) Anti-apoptotic	(1) Cx26 and KCNQ1; (2) ROS and cas-3	[187, 188]
	*Scutellaria baicalensis* Georgi	Baicalein		(1) HCs protection; (2) Anti-oxidant; (3) Anti-apoptotic	(1) Auditory center; (2) HCs; (3) PARP and cas-3; (4) ROS	[190]
	*Panax ginseng* C.A. Mey	Ginsenoside Rb1		(1) Anti-oxidant; (2) Anti-apoptotic	(1) ROS; (2) JNK, Bcl-xL, Bax, Cyt-c, cas-3, PARP	[195]
		Ginsenoside compound K				
	*Stephania tetrandra* S. Moore	Tetrandrine		(1) Blocks Ca^2+^ channels; (2) Neuroprotection; (3) HCs protection	(1) Ca^2+^ channels; (2) Auditory nerve; (3) OHCs	[200]
	*Allium sativum* L	Allicin		(1) Anti-oxidant; (2) Neuroprotection; (3) Anti-apoptotic; (4) Mitochondrial protection	(1) SGN mitochondria; (2) Bax, cas-9,3, p53, Cyt-c; (3) MDA, SOD	[202]
		SAMC				
		DD				
		SAC				
	Leaf of *Ginkgo biloba* L	EGb 761	/	(1) Anti-oxidant; (2) Neuroprotection; (3) Anti-apoptotic; (4) Anti-inflammatory; (5) Mitochondrial protection	(1) Peripheral and central auditory systems; (2) amyloid-beta, cas-3; (3) ROS, NO; (4) Neural stem cells; (5) IL-1β, IL-6, TNF-α and COX-2; (6) HSP-70, HSF-1; (7) SIRT1	[205–214]
		Ginkgolide B				
	Peel of *Punica granatum* L	/	/	(1) Anti-oxidant; (2) Anti-apoptotic	(1) ROS; (2) 4-HNE, PNUTS, p53 and cas-3; (3) PP1 and MDM2	[220, 221]
	*Glycine max* (L.) Merr	β-Conglycinin	/	(1) Increase cochlear blood flow; (2) Anti-oxidant; (3) mitochondrial protection	(1) Cochlear vessels; (2) mtDNA	[222, 223]
		Lecithin				
Drugs related to liver meridian in herbal medicines	*Salvia mitiorrhiza* Bge	Tanshinone		(1) Anti-oxidant; (2) Anti-apoptotic	(1) HCs; (2) ROS	[224–226]
	*Curcuma longa* L	Curcumin		(1) HCs protection; (2) Anti-oxidant	(1) OHCs; (2) 4-HNE; (3) HO-1	[227]
	*Uncaria rhynchophylla* (Miq.) Miq.ex Havil	Hydrophilic chemotype carboxy alkyl esters	/	(1) Anti-oxidant; (2) Anti-inflammatory	(1) OHCs; (2) Auditory nerve;	[230]
	Peel of *Erythrina variegata* L	Erythrivarine N	/	Neuroprotection	/	[231, 232]
		Erythrivarine T	/			

### Drugs related to kidney meridian in herbal medicines

According to TCM theory, the kidney opens into the ear. Compared with the general population, patients with chronic renal failure are more likely to also experience sensorineural hearing loss. The study found that the incidence rate is 77% [163]. Because the physiological mechanisms of the cochlea and kidney are comparable [164], there is hope for treating hearing loss via the kidney.

#### *Polygonum multiflorum* Thunb. (He Shou Wu)

*Polygonum multiflorum* Thunb., its flavor is bitter, sweet, and astringent, and its nature is slightly warm, belonging to the liver and kidney meridians. It is frequently used as a tonic or anti-aging agent in several regions of Asia and is frequently utilized in clinics to treat a wide range of chronic conditions [165]. 2,3,4’,5-Tetrahydroxystilbene-2-O-β-D-glucoside (THSG), the main component of *Polygonum multiflorum* Thunb. It was found that THSG has a similar free radical scavenging capacity to ascorbic acid, and can prevent H_2_O_2_-induced autophagy in UB/OC-2 cells and inhibit H_2_O_2_-induced apoptosis through the mitochondrial pathway. The mRNA expression of HO-1 and NQO1 increased as a result of Nrf2 translocation to the nucleus, suggesting that THSG can enhance antioxidant defense against oxidative stress and play a role in hearing preservation [166]. Additionally, it has been discovered that THSG can reduce the ototoxicity of gentamicin-induced UB/OC-2 cochlear cells by regulating autophagy signal of Sesn2/AMPK/mTOR pathway [167]. However, *Polygonum multiflorum* Thunb. should not be consumed in large doses over an extended period of time due to the possibility of liver damage.

#### *Drynaria fortunei* (Kunze) J.Sm. (Gu Sui Bu)

*Drynaria fortunei* (Kunze) J.Sm., its flavor is bitter and warm in nature, and belongs to the liver and kidney meridians. It has been used to treat fractures with good results. It is frequently combined with *Rehmannia glutinosa* Libosch. and *Cornus officinalis* Sieb. et Zucc when treating tinnitus and hearing loss due to by kidney failure. Studies have shown that *Drynaria fortunei* (Kunze) J.Sm. can protect against the ototoxicity of aminoglycosides and streptomycin in guinea pig cochlear HCs, hence defending hearing [168, 169]. The flavanoid component in *Drynaria fortunei* (Kunze) J.Sm. may be its active ingredient. It can protect the ototoxicity of animals caused by gentamicin, and the damaged both IHCs and OHCs recover well without hindering the efficacy of gentamicin [170].

#### *Cornus officinalis* Sieb.et Zucc. (Shan Zhu Yu)

*Cornus officinalis* Sieb.et Zucc., it has a sour and astringent flavor, is slightly warm in nature, and belongs to the liver and kidney meridians. It is one of the ingredients in the Erlong Zuoci which is a traditional deafness prescription (calcined magnet, *Rehmannia glutinosa* Libosch., *Cornus officinalis* Sieb.et Zucc., *Alisma orientale* (Sam.) Juzep., *Poria cocos* (Schw.) Wolf, *Paeonia suffruticosa* Andr., *Dioscorea opposita* Thunb., *Bupleurum chinense* DC.), this prescription has been shown to elimination of DNA damage in hydrogen peroxide-induced auditory HCs, blocks up-regulation of cellular senescence proteins p21 and p-p53, increases p-ERK (ERK, extracellular regulated protein kinase) expression, reduces p-STAT3 (STAT3 activation is closely related to cancer) expression [171], and protects cochlear cells from ototoxicity caused by gentamicin [172]. Ursolic acid, the primary active ingredient in *Cornus officinalis* Sieb. et Zucc., can inhibit lipid peroxidation in a dose-dependent manner and activate the antioxidant enzymes CAT and GPx to prevent damage to auditory HCs caused by hydrogen peroxide [173].

#### *Rehmannia glutinosa* Libosch. (Di Huang)

*Rehmannia glutinosa* Libosch., its flavor is sweet, slightly warm in nature, and belongs to the meridians of the liver and kidneys. It nourishes the liver and kidneys, and is used to treat tinnitus and hearing loss due to liver and kidney deficiency. It is often used in conjunction with *Cornus officinalis* Sieb.et Zucc. and *Dioscorea opposita* Thunb., it is the monarchical medicine of the six-flavor *Rehmannia* Decoction, a common tonic. And it has been shown that six-flavor *Rehmannia* Decoction could effectively reduce the ototoxic effect of gentamicin on the inner ear of guinea pigs [174]. Ethanol extract from the steamed root of *Rehmannia glutinosa* Libosch. can protect auditory cells from the ototoxicity of cisplatin in a dose-dependent manner, and its pharmacological effects may be related to the activation of intracellular anti-oxidant enzymes and inhibition of lipid peroxidation [175, 176].

#### *Sesamum indicum* L. (Hei Zhi Ma)

*Sesamum indicum* L., has a sweet flavor, is flat in nature, and belongs to the liver, kidney, and large intestine meridians. This is a great food to supplement the body’s nutrition. Rich in *Sesamum indicum* L. oil and sesamin, *Sesamum indicum* L. oil decreased the hearing threshold changes caused by clicks and 8,16-kHz tone bursts in NIHL mice and it improved hearing impairment. Additionally, research on the hearing-related gene Tecta revealed that sesamin plays a crucial protective role in auditory function by shielding auditory HCs from damage and reversing it [177].

#### *Tripterygium wilfordii* Hook. f. (Lei Gong Teng)

*Tripterygium wilfordii* Hook. f., which has a bitter and pungent flavor, is cold in nature, highly poisonous, and belongs to the liver and kidney meridians. It is mainly used to treat rheumatoid arthritis. One of the key components of *Tripterygium wilfordii* Hook. f., Celastrol, is a pentacyclic triterpenoid compound that inhibits ototoxicity by activating heat shock protein (HSP) 32/heme oxygenase-1 (HO-1) and NF-E2-related factor-2 (Nrf2, a crucial component of the cellular oxidative stress response) in HCs in neomycin-treated mice [178]. Recent studies have shown that celastrol can significantly increases the activity and proliferation of inner ear stem cells, stimulates the differentiation of inner ear stem cells into neuron-like cells, and enhances neural excitability and electrophysiological activity. These results may be associated with the upregulation of the Atoh1 gene, an important positive regulator of sensory cell differentiation in the inner ear [179]. However, due to high level of toxicity of *Tripterygium wilfordii* Hook. f., users frequently report unpleasant side effects like diarrhea, indigestion, nausea, abdominal pain, and upper respiratory infections [180]. Consequently, the use of *Tripterygium wilfordii* Hook. f. is extremely risky.

#### *Poria cocos* (Schw.) Wolf (Fu Ling)

*Poria cocos* (Schw.) Wolf, its flavor is sweet and mild, is flat in nature, returns to the heart, lungs, spleen and kidney meridians. Studies have shown that the decoction of *Poria cocos* (Schw.) Wolf can prevent kanamycin-induced ototoxicity in guinea pigs [181].

### Drugs related to lung meridian in herbal medicines

There are many related theories of lung and hearing loss in TCM, “lung main Qi, one’s Qi passes through the ear, so can hear sound”, and some hearing loss is triggered by a cold, and cold is also associated with the dysregulation of lung function, so it is believed that lung and hearing loss have a close relationship. In addition, *Glycine max* (L.) Merr. and peels of *Punica granatum* L. belong to the large intestine meridian rather than the lung meridian. However, TCM theory holds that there is an exterior-interior relationship between the lung and the large intestine, and that the dysfunction of the large intestine also cause lung diseases. There is currently pertinent research showing the intimate connection between the large intestine and the lung [182]. Therefore, these herbal medicines can also be incorporated into the lung meridian for description.

#### *Pueraria lobata* (Willd.) Ohwi (Ge Gen)

*Pueraria lobata* (Willd.) Ohwi, its flavor is sweet and pungent, cool in nature, returns to the spleen, stomach, and lung meridians, and is mostly used for the treatment of cardiovascular and cerebrovascular diseases and hearing loss [183]. Rats' hearing threshold and hemorheology items significantly improved following treatment with *Pueraria lobata* (Willd.) Ohwi, which had a good preventative impact on senile diseases in rats [184]. The main active ingredient of *Pueraria lobata* (Willd.) Ohwi is puerarin, an isoflavone compound. Studies have shown that puerarin treatment can significantly improve the threshold of auditory brainstem response (ABR) in NIHL mice. It was discovered through research on the cochlear nucleus in the central auditory pathway that it reduced the expression of the protein kinase C gamma subunit (PKCγ) after noise exposure and increased the decrease of GABAB receptor 1 (GABABR1) and GABABR2; this may be one of the pharmacological mechanisms of its effect [185].

#### *Astragalus membranaceus* (Fisch.) Bge. (Huang Qi)

*Astragalus membranaceus* (Fisch.) Bge., its flavor is sweet, slightly warm in nature, and returns to the meridians of the spleen and lungs. It is often combined with *Panax ginseng* C.A. Mey., *Cimicifuga foetida* L., and *Bupleurum chinense* DC., such as Buzhong Yiqi Decoction, a TCM formula for deafness and tinnitus caused by spleen deficiency. *Astragalus membranaceus* (Fisch.) Bge. is a natural anti-oxidant. Injections of *Astragalus membranaceus* (Fisch.) Bge. can promote the recovery of sudden hearing loss [186]. *Astragalus membranaceus* (Fisch.) Bge. was able to significantly reduce ABR defects and SV damage caused by impulse noise, and reduce the shift of expression of Cx26 and KCNQ1 in SV [187]. Astragaloside IV, is the main active component of *Astragalus membranaceus* (Fisch.) Bge., has been found to significantly reduce ABR deficiency in impulsive noise-induced hearing loss and reduce ROS and the expression of cas-3 by intragastric administration of astragaloside IV to guinea pigs [188].

#### *Scutellaria baicalensis* Georgi (Huang Qin)

*Scutellaria baicalensis* Georgi, its flavor is bitter, cold in nature, belongs to the meridians of the lungs, gallbladder, spleen, large intestine and small intestine. It is commonly used to remove heat and harmful substances in the body, promote blood circulation and remove blood stasis, and induce diuresis to reduce edema [189]. A study found that in the NIHL mice model, *Scutellaria baicalensis* Georgi extract significantly reduced the threshold shift, central auditory function impairment and cochlear function defect, indicating that *Scutellaria baicalensis* Georgi can protect the auditory function of animals [190]. Baicalein, is a flavonoid molecule, is the product's main ingredient for preventing hearing loss. After biotransformation, a higher concentration of baicalein can effectively stimulate the recovery of zebrafish HCs and improve hearing in mice [191]. Baicalein was able to down-regulate the activation of poly (ADP-ribose) polymerase (PARP, a DNA repair enzyme and is also a cleavage substrate for caspases) and cas-3 that were increased by gentamicin treatment. Baicalin attenuated gentamicin-induced cochlear HCs ototoxicity, and this inhibitory effect may be mediated through regulation of ROS production, mitochondrial depolarization, and activation of cas-3 and PARP [192].

#### *Panax ginseng* C.A. Mey. (Ren Shen)

*Panax ginseng* C.A. Mey., its flavor is sweet and slightly bitter, is slightly warm in nature, and belongs to the meridians of the spleen, lungs, heart and kidneys. It is a key drug for the prevention of critical diseases and has been used for thousands of years in TCM to treat diseases of Qi deficiency. Red *ginseng* is a steamed product of *Panax ginseng* C.A. Mey. With its anti-ROS properties, Korean red *ginseng* can prevent hearing loss induced by cisplatin and the mitochondrial toxin 3-Nitropropionic Acid (3-NP) [193, 194]. Studies have found that ginsenoside Rb1, one of the active components of *Panax ginseng* C.A. Mey., can reduce hearing loss in rats caused by gentamicin by attenuating the production of ROS and inhibiting cell apoptosis [195]. Ginsenoside compound K may be the another active ingredient in *Panax ginseng* C.A. Mey. that can treat hearing loss [196]. However, long-term and high-dose (500 mg/kg) treatment of red *ginseng* may cause excitatory side effects and aggressive behavior in mice [197].

In an open-label randomized controlled trial of 61 patients, *Panax ginseng* C.A. Mey. was found to improve tinnitus symptoms and mental health in people with chronic tinnitus [198]. Co-treatment of N-acetylcysteine (NAC) and *Panax ginseng* C.A. Mey. could reduce noise-induced temporary threshold shift in textile workers exposed to occupational noise [199]. The results of these studies, which were limited to animal models and a small population, can only be considered preliminary and more research are needed to determine the pharmacological effects of *Panax ginseng* C.A. Mey. in the treatment of hearing loss and its active constituents.

#### *Stephania tetrandra* S. Moore (Fang Ji)

*Stephania tetrandra* S. Moore, its flavor is bitter, cold in nature, belongs to the meridians of the bladder and lungs. It is used to treat rheumatoid arthritis, edema in the lower extremities, and dysuria. Its main chemical isolate is tetrandrine, which significantly attenuates NIHL in CBA/CaJ mice, which by blocking Ca^2+^ channels, prevents and protects neurotrauma, prevents damage to OHCs and synapses, and has durable protection against noise exposure effect [200].

#### *Allium sativum* L. (Da Suan)

*Allium sativum* L., which has a spicy flavor, warm in nature, returns to the spleen, stomach and lungs meridians. It is a common excipient in dishes and has the functions of killing parasites and detoxifying. Male Wistar rats treated with *Allium sativum* L. diet showed a substantial reduction in the gentamicin-induced rise in hearing threshold [201]. Allicin, contained in *Allium sativum* L., protects SGN mitochondria from damage and reduces the release of Cyt-c, as well as significantly reducing the expression of cisplatin-activated pro-apoptotic factors, including Bax, cas-9, cas-3 and p53. It can also decrease malondialdehyde (MDA) levels while increasing SOD levels [202]. *Allium sativum* L. has three active compounds: S-Allylmercaptocysteine (SAMC), Diallyl Disulfide (DD), and S-Allylcysteine (SAC), all of which are capable of lowering the ototoxicity of aminoglycosides. SAMC and DD seem to be more prevalent than SAC [203].

#### Leaf of *Ginkgo biloba* L. (Yin Xing Ye)

*Ginkgo biloba* L., with its sweet, bitter, and astringent flavor, is neutral in nature, returning to the heart and lungs meridians. *Ginkgo biloba* L., a popular anti-oxidant herbal remedy with anti-cancer and anti-aging properties, has been used to cure illnesses for more than 5,000 years. The active ingredient in *Ginkgo biloba* L. for the treatment of hearing loss is *Ginkgo biloba* L. extract (EGb 761), which consists of about 80 different compounds. EGb 761 was able to prevent NIHL in guinea pigs, and there have higher amplitudes of the acoustic nerve potentials in EGb 761-treated animals than in untreated animals [204]. EGb 761 has a dramatic neuroplastic effect on auditory processing at the peripheral and central levels [205]. It protects neuronal mitochondrial ATP synthesis under oxidative stress, improves neuronal energy metabolism [206, 207], and achieves neuroprotection through anti-apoptotic properties [208–210]. Additionally, there were some beneficial effects from the use of *Ginkgo biloba* L. extracts in the treatment of cells and neural stem cell proliferation [211]. It also has a certain effect on hearing loss caused by inflammation. Studies have confirmed that it can inhibit the expression of IL-1β, IL-6, TNF-α and cyclooxygenase 2 (COX-2, which plays an important role in apoptosis and tumorigenesis), and increase the expression of HSP-70 and heat shock factor 1 (HSF-1) values in the rat cochlea [212]. By inhibiting mitochondrial apoptosis and ERK, its combination with cilostazol protects rats from cisplatin-induced cochlear and vestibular dysfunction [213]. EGb 761 also prevents hearing loss caused by 3-NP in a rat model of acute ototoxicity, which may be related to activation of the expression of Sirtuin 1 (SIRT1) [214]. Ginkgolide B (GB), the main component of *Ginkgo biloba* L. extract, exerts a protective effect on cisplatin-induced ototoxicity in rats without weakening the antitumor activity of cisplatin [215, 216].

By investigating 56 patients with unilateral sudden sensorineural hearing loss, it was found that the pure-tone audiometric gains of patients receiving *Ginkgo biloba* diterpene lactone supplementation were significantly greater than those receiving methylprednisolone alone, especially those with severe hearing loss [217]. *Ginkgo biloba* L. extract may be effective as an adjunct to corticosteroids in the initial treatment of moderate to severe sudden sensorineural hearing loss [218]. According to the results of a multicenter, randomized, double-blind clinical trial, the combination of steroids and EGb761 for initial treatment of hearing loss did not show better pure tone thresholds than patients treated with steroids alone. However, patients treated with the combination showed a significant improvement in speech discrimination [219]. Studies of the hearing protection effects of *Ginkgo biloba* L. still require large cohort studies, as investigations of *Ginkgo biloba* L. extracts have only been performed in animal models and in a small number of groups.

#### Peel of *Punica granatum* L. (Shi Liu Pi)

*Punica granatum* L., its flavor is sour and astringent, is warm in nature, and belongs to the meridian of the large intestine. The peel of *Punica granatum* L. is commonly used to treat diarrhoea. The peel of *Punica granatum* L. extract has a protective effect on gentamicin-induced ototoxicity in Wistar albino rats and can reduce the level of cochlear ROS [220], and it can also reverse the 4-hydroxynonenal (4-HNE, a representative substance in cellular lipid peroxidation), inhibits the expression of protein phosphatase 1 nuclear targeting subunit (PNUTS), p53 and cas-3, and promotes the decreased expression of protein phosphatase1 (PP1) and MDM2 (oncogene) in the cochlea [221].

#### *Glycine max* (L.) Merr. (Da Dou)

*Glycine max* (L.) Merr. is a very common meal that contains a variety of proteins. β-Conglycinin (β-CG) is one of the main proteins in *Glycine max* (L.) Merr., which has anti-obesity and anti-atherosclerosis effects. Hearing impairment was protected in mice fed β-CG, it can increase cochlear blood flow and has anti-oxidative stress effects [222]. *Glycine max* (L.) Merr. is also rich in lecithin. Studies have found that lecithin can significantly reduce the specific deletion of mtDNA in the cochlea, protect mitochondrial function, and thus play a role in hearing protection [223].

### Drugs related to liver meridian in herbal medicines

Emotion and hearing loss are closely related to one another. According to the TCM theory, the liver is associated with irritability, the liver is stored in blood and the kidney is stored in essence, and since essence and blood share the same origin, liver and kidney dysfunctions are also closely related to hearing loss. Many hearing loss patients also experience excessive anger and hyperactivity of liver yang.

#### *Salvia mitiorrhiza* Bge. (Dan Shen)

*Salvia miltiorrhiza* Bge., its flavor is bitter, slightly cold in nature, returns to the heart and liver meridians. It is commonly used to treat blood stasis syndrome. It can reduce the increase of hearing threshold, alleviate cell apoptosis, reduce the oxidative damage of cochlear ultrastructure such as SV and SGN, and has a protective effect on guinea pig ototoxicity caused by gentamicin and cisplatin [224, 225]. Tanshinone is one of its active ingredients, and in vitro studies have shown that it significantly reduces the formation of free radicals and lipid peroxidation induced by gentamicin, reduces the increase in hearing threshold induced by kanamycin in a dose-dependent manner, and reduces HCs loss. And its role of hearing protection without affecting the serum level and anti-bacterial effect of the drug [226].

#### *Curcuma longa* L. (Jiang Huang)

*Curcuma longa* L., its flavor is pungent and bitter, warm in nature, returns to the liver and spleen meridians. It is commonly used to treat blood stasis syndrome and rheumatic pain. Curcumin is one of its main active ingredients, which can increase the survival rate of OHCs, reduce the expression of 4-HNE, and increase HO-1 expression, attenuates cisplatin-induced hearing loss [227], and studies have shown that curcumin attenuates all stages of head and neck squamous cell carcinoma progression (survival, proliferation), thus being an effective adjuvant to cisplatin and protecting against ototoxicity effect [228]. A synthetic analog of curcumin called EF-24, which has a better bioavailability than curcumin, can also be used as a cancer treatment and has a protective effect against cisplatin-induced ROS generation in zebrafish inner ear tissue [229].

#### *Uncaria rhynchophylla* (Miq.) Miq.ex Havil (Gou Teng)

*Uncaria rhynchophylla* (Miq.) Miq.ex Havil, its flavor is sweet, cool in nature, returns to the liver and pericardium meridians. It is commonly used to treat epileptic seizures. It has been studied that treatment with a hydrophilic chemotype carboxy alkyl esters of *Uncaria tomentosa* almost completely restored noise-exposed OHCs function and limited the extent of cell loss, inhibiting the loss of neural sensitivity to pure tone stimulation [230], which may be related to its association with cytoprotective properties, including enhanced cellular DNA repair, anti-oxidant and anti-inflammatory effects.

#### Peel of *Erythrina variegata* L. (Hai Tong Pi)

The peel of *Erythrina variegata* L., has a bitter and acrid flavor, is flat in nature, and belongs to the liver meridian. It is commonly used to treat lower body rheumatoid arthritis. Erythrivarines J-N and erythrivarines O-Z are alkaloids isolated from the peel of *Erythrina variegata* L., and studies have shown that erythrivarine N and erythrivarine T have neuroprotective effects on neomycin-induced hearing loss [231, 232].

**Fig. 2:**
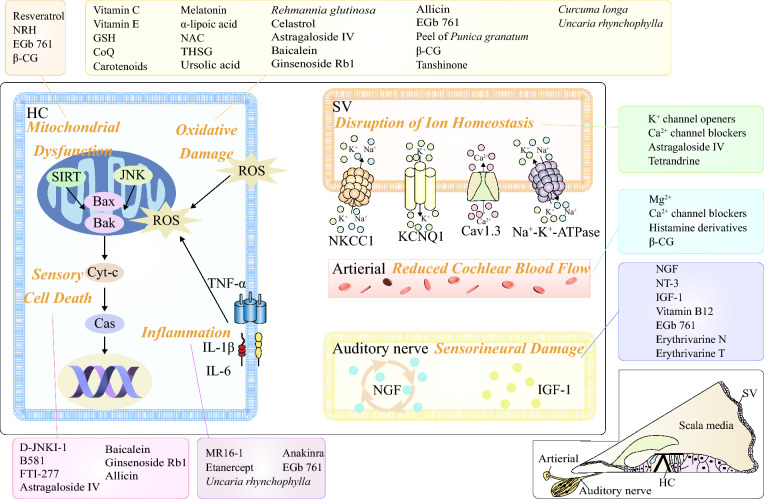
Pathophysiological mechanisms of age-related hearing loss and potential drug candidates. *HC* hair cell, *SV* stria vascularis, *SIRT* sirtuin family, *JNK* c-Jun N-terminal kinase, *Cyt-c* cytochrome c, Cas, Bax, *Bak* pro-apoptotic factors, *ROS* reactive oxygen species, *TNF-α* tumor necrosis factor α, *IL* interleukin, *NKCC1* Na^+^-K^+^-2Cl^−^ co-transporter isoform 1, *KCNQ1* potassium voltage-gated channel subfamily q member 1, *Cav 1.3* voltage-gated calcium channel, *NGF* nerve growth factor, *IGF-1* insulin-like growth factor 1, *NRH* the reduced form of nicotinamide ribose, *EGb 761* active ingredient in Ginkgo biloba L., *β-CG* β-Conglycinin, *GSH* glutathione, *CoQ* coenzyme Q, *NAC* N-acetylcysteine, *THSG* 2,3,4’,5-Tetrahydroxystilbene-2-O-β-D-glucoside, *NT-3* neurotrophic factor-3, *D-JNKI-1* a cell-penetrating peptide that blocks JNK signaling, B581, FTI-277: inhibition of RAS, which is an upstream activator of the JNK pathway; *MR16-1* anti-IL-6 receptor antibody

The pathophysiological mechanisms responsible for hearing loss and the corresponding herbal remedies and contemporary medications are shown in Fig. 2.

## Conclusions

ARHL not only affects the independent living ability of elderly people, but may also lead to a number of mental illnesses in elderly patients. However, there are presently no efficient therapeutic drugs for ARHL. This review suggests that the main causes of ARHL are genetic and environmental factors. Currently, research on the pathology of ARHL mainly focuses on oxidative damage, mitochondrial dysfunction, inflammation, cochlear blood flow, ion homeostasis and other aspects. Anti-oxidants, mitochondrial function regulators, anti-inflammatory drugs, vasodilators, K^+^ channel openers, Ca^2+^ channel blockers, JNK inhibitors, and nerve growth factor/neurotrophin all contribute to hearing protection. Healthy living habits, such as avoiding the use of ototoxic drugs, avoiding noise exposure, not smoking, consuming moderate amounts of alcohol, exercising in moderation, ensuring quality sleep, and eating more anti-oxidant foods also help for preventing ARHL. ARHL patients can also improve speech recognition in noisy environments by training [233]. Moreover, cell replacement therapy may also develop into a novel form of therapy [234]. There are many studies to treat ARHL by regenerating functional HCs or SGN in the old inner ear with adult stem cells, embryonic stem cells and induced pluripotent stem cells [27].

In traditional Chinese medicine, herbal medicines belong to the meridians of kidney, lung, and liver have good hearing protection. In this review, we ultimately found 7 herbal medicines belonging to the kidney meridian, 9 to the lungs meridian, and 4 to the liver meridian. Thus, Chinese herbal medicines are an important source of potential anti-ARHL drugs. In addition to the herbal remedies already mentioned, there are numerous other herbs for treating hearing loss and tinnitus that have been used in China for thousands of years but have not yet undergone thorough research, such as *Phellodendron chinense* Schneid, *Coptis chinensis* Franch., *Alisma orientale* (Sam.) Juzep., *Paeonia suffruticosa* Andr., *Bupleurum chinense* DC., etc. On the contrary, some herbal medicines lead to the occurrence of NIHL, such as *Ephedra sinica* Stapf [235], *Areca catechu* L. [236], and cinnabar [237]. The mechanism of the hearing protection effect of these herbal medicines and their active constituents needs to be investigated further. There are also some research findings indicate that acupuncture can improve local blood circulation, promote blood flow to the ear, increase blood oxygen supply, decrease blood viscosity, regulate the inflammatory response, improve lymph circulation, and enhance the excitability of auditory nerve [238]. Intervention combining acupuncture with medicine comprehensive treatment had more efficacious in treating sudden sensorineural hearing loss [238].

This review summarizes the potential therapeutic effects of herbal remedies on ARHL to date and the pharmacological mechanisms involved. It provides researchers with an inspiration: the focus of treatment for ARHL can be on herbal remedies. However, these herbal remedies still have some limitations: the active ingredients in the herbs are less stable and can be easily affected by temperature, humidity, light, and other factors; currently, there is a lack of scientific and reasonable clinical research to prove the efficacy of traditional Chinese medicine; it is difficult to fully determine the active ingredient of herbal remedies for ARHL due to its complex composition. These issues are in urgent need of further future research.

## Data Availability

Not applicable.

## Abbreviations

ARHL: Age-related hearing loss
AC: Air conduction
BC: Bone conduction
WHO: World Health Organization
TCM: Traditional Chinese medicine
HCs: Hair cells
OHCs: Outer hair cells
IHCs: Inner hair cells
SV: Stria vascularis
EP: Endocochlear potential
SL: Spiral ligament
SGNs: Spiral ganglion neurons
GWAS: Genome-wide association study
ROS: Reactive oxygen species
SOD2: Superoxide dismutase 2
TNF: Tumor necrosis factor
IL-1: Interleukin 1
CAT: Catalase
GPx: Glutathione peroxidase
GR: Glutathione reductase
GSH: Glutathione
NAC: N-acetylcysteine
NIHL: Noise-induced hearing loss
Cyt-c: Cytochrome c
JNK: C-Jun N-terminal kinase
NGF: Nerve growth factor
IGF-1: Insulin-like growth factor 1
THSG: 2,3,4’,5-Tetrahydroxystilbene-2-O-β-D-glucoside
HSP: Heat shock protein
HO-1: Heme oxygenase-1
ABR: Auditory brainstem response
SAMC: S-Allylmercaptocysteine
DD: Diallyl disulfide
SAC: S-Allylcysteine
GB: Ginkgolide B

## References

[1] Chadha S, Kamenov K, Cieza A (2021). The world report on hearing, 2021. Bull World Health Organ.

[2] Collins JG (1997). Prevalence of selected chronic conditions: United States, 1990–1992. Vital Health Stat.

[3] Feng L, Wu D, Lin J, Li Y, Zhao Y, Zhang P, Yao Y, Fu S (2022). Associations between age-related hearing loss, cognitive decline, and depression in Chinese centenarians and oldest-old adults. Ther Adv Chronic Dis.

[4] Lin FR, Metter EJ, O'Brien RJ, Resnick SM, Zonderman AB, Ferrucci L (2011). Hearing loss and incident dementia. Arch Neurol.

[5] Man J, Chen H, Zhang T, Yin X, Yang X, Lu M (2021). Global, regional, and national burden of age-related hearing loss from 1990 to 2019. Aging (Albany NY).

[6] Schuknecht HF, Gacek MR (1993). Cochlear pathology in presbycusis. Ann Otol Rhinol Laryngol.

[7] Welsh LW, Welsh JJ, Healy MP (1985). Central presbycusis. Laryngoscope.

[8] Bredberg G (1968). Cellular pattern and nerve supply of the human organ of Corti. Acta Otolaryngol.

[9] Wu PZ, Liberman LD, Bennett K, de Gruttola V, O'Malley JT, Liberman MC (2019). Primary neural degeneration in the human cochlea: evidence for hidden hearing loss in the aging ear. Neuroscience.

[10] Gates GA, Mills JH (2005). Presbycusis. Lancet.

[11] Salt AN, Melichar I, Thalmann R (1987). Mechanisms of endocochlear potential generation by stria vascularis. Laryngoscope.

[12] Wangemann P (2006). Supporting sensory transduction: cochlear fluid homeostasis and the endocochlear potential. J Physiol.

[13] Hibino H, Nin F, Tsuzuki C, Kurachi Y (2010). How is the highly positive endocochlear potential formed? The specific architecture of the stria vascularis and the roles of the ion-transport apparatus. Pflugers Arch.

[14] Fernandez KA, Jeffers PW, Lall K, Liberman MC, Kujawa SG (2015). Aging after noise exposure: acceleration of cochlear synaptopathy in "recovered" ears. J Neurosci.

[15] Kujawa SG, Liberman MC (2015). Synaptopathy in the noise-exposed and aging cochlea: primary neural degeneration in acquired sensorineural hearing loss. Hear Res.

[16] Keithley EM, Feldman ML (1979). Spiral ganglion cell counts in an age-graded series of rat cochleas. J Comp Neurol.

[17] Keithley EM, Ryan AF, Woolf NK (1989). Spiral ganglion cell density in young and old gerbils. Hear Res.

[18] Furman AC, Kujawa SG, Liberman MC (2013). Noise-induced cochlear neuropathy is selective for fibers with low spontaneous rates. J Neurophysiol.

[19] Viana LM, O'Malley JT, Burgess BJ, Jones DD, Oliveira CA, Santos F, Merchant SN, Liberman LD, Liberman MC (2015). Cochlear neuropathy in human presbycusis: confocal analysis of hidden hearing loss in post-mortem tissue. Hear Res.

[20] Nayagam BA, Muniak MA, Ryugo DK (2011). The spiral ganglion: connecting the peripheral and central auditory systems. Hear Res.

[21] Vermiglio AJ (2016). On diagnostic accuracy in audiology: central site of lesion and central auditory processing disorder studies. J Am Acad Audiol.

[22] Lin FR, Ferrucci L, An Y, Goh JO, Doshi J, Metter EJ, Davatzikos C, Kraut MA, Resnick SM (2014). Association of hearing impairment with brain volume changes in older adults. Neuroimage.

[23] Jafari Z, Kolb BE, Mohajerani MH (2021). Age-related hearing loss and cognitive decline: MRI and cellular evidence. Ann N Y Acad Sci.

[24] Wang M, Zhang C, Lin S, Wang Y, Seicol BJ, Ariss RW, Xie R (2021). Biased auditory nerve central synaptopathy is associated with age-related hearing loss. J Physiol.

[25] Bowl MR, Dawson SJ (2019). Age-related hearing loss. Cold Spring Harb Perspect Med.

[26] Keithley EM (2020). Pathology and mechanisms of cochlear aging. J Neurosci Res.

[27] Wang J, Puel JL (2020). Presbycusis: an update on cochlear mechanisms and therapies. J Clin Med.

[28] Altschuler RA, Dolan DF, Halsey K, Kanicki A, Deng N, Martin C, Eberle J, Kohrman DC, Miller RA, Schacht J (2015). Age-related changes in auditory nerve-inner hair cell connections, hair cell numbers, auditory brain stem response and gap detection in UM-HET4 mice. Neuroscience.

[29] Ter Haar G, de Groot JC, Venker-van Haagen AJ, van Sluijs FJ, Smoorenburg GF (2009). Effects of aging on inner ear morphology in dogs in relation to brainstem responses to toneburst auditory stimuli. J Vet Intern Med.

[30] Burianova J, Ouda L, Syka J (2015). The influence of aging on the number of neurons and levels of non-phosporylated neurofilament proteins in the central auditory system of rats. Front Aging Neurosci.

[31] Azaiez H, Booth KT, Ephraim SS, Crone B, Black-Ziegelbein EA, Marini RJ, Shearer AE, Sloan-Heggen CM, Kolbe D, Casavant T, Schnieders MJ, Nishimura C, Braun T, Smith RJH (2018). Genomic landscape and mutational signatures of deafness-associated genes. Am J Hum Genet.

[32] Hereditary Hearing Loss Homepage. https://hereditaryhearingloss.org/. Accessed 2 Jan 2023.

[33] Lavinsky J, Ge M, Crow AL, Pan C, Wang J, Salehi P, Myint A, Eskin E, Allayee H, Lusis AJ, Friedman RA (2016). The genetic architecture of noise-induced hearing loss: evidence for a gene-by-environment interaction. G3 (Bethesda).

[34] Fransen E, Bonneux S, Corneveaux JJ, Schrauwen I, Di Berardino F, White CH, Ohmen JD, Van de Heyning P, Ambrosetti U, Huentelman MJ, Van Camp G, Friedman RA (2015). Genome-wide association analysis demonstrates the highly polygenic character of age-related hearing impairment. Eur J Hum Genet.

[35] Van Laer L, Van Eyken E, Fransen E, Huyghe JR, Topsakal V, Hendrickx JJ, Hannula S, Maki-Torkko E, Jensen M, Demeester K, Baur M, Bonaconsa A, Mazzoli M, Espeso A, Verbruggen K, Huyghe J, Huygen P, Kunst S, Manninen M, Konings A, Diaz-Lacava AN, Steffens M, Wienker TF, Pyykko I, Cremers CW, Kremer H, Dhooge I, Stephens D, Orzan E, Pfister M, Bille M, Parving A, Sorri M, Van de Heyning PH, Van Camp G (2008). The grainyhead like 2 gene (GRHL2), alias TFCP2L3, is associated with age-related hearing impairment. Hum Mol Genet.

[36] Lin YH, Wu CC, Hsu CJ, Hwang JH, Liu TC (2011). The grainyhead-like 2 gene (GRHL2) single nucleotide polymorphism is not associated with age-related hearing impairment in Han Chinese. Laryngoscope.

[37] Huebner AK, Maier H, Maul A, Nietzsche S, Herrmann T, Praetorius J, Hubner CA (2019). Early hearing loss upon disruption of Slc4a10 in C57BL/6 Mice. J Assoc Res Otolaryngol.

[38] Potter PK, Bowl MR, Jeyarajan P, Wisby L, Blease A, Goldsworthy ME, Simon MM, Greenaway S, Michel V, Barnard A, Aguilar C, Agnew T, Banks G, Blake A, Chessum L, Dorning J, Falcone S, Goosey L, Harris S, Haynes A, Heise I, Hillier R, Hough T, Hoslin A, Hutchison M, King R, Kumar S, Lad HV, Law G, MacLaren RE, Morse S, Nicol T, Parker A, Pickford K, Sethi S, Starbuck B, Stelma F, Cheeseman M, Cross SH, Foster RG, Jackson IJ, Peirson SN, Thakker RV, Vincent T, Scudamore C, Wells S, El-Amraoui A, Petit C, Acevedo-Arozena A, Nolan PM, Cox R, Mallon AM, Brown SD (2016). Novel gene function revealed by mouse mutagenesis screens for models of age-related disease. Nat Commun.

[39] Girotto G, Morgan A, Krishnamoorthy N, Cocca M, Brumat M, Bassani S, La Bianca M, Di Stazio M, Gasparini P (2019). Next Generation sequencing and animal models reveal SLC9A3R1 as a new gene involved in human age-related hearing loss. Front Genet.

[40] Van Eyken E, Van Laer L, Fransen E, Topsakal V, Lemkens N, Laureys W, Nelissen N, Vandevelde A, Wienker T, Van De Heyning P, Van Camp G (2006). KCNQ4: a gene for age-related hearing impairment?. Hum Mutat.

[41] Van Laer L, DeStefano AL, Myers RH, Flothmann K, Thys S, Fransen E, Gates GA, Van Camp G, Baldwin CT (2002). Is DFNA5 a susceptibility gene for age-related hearing impairment?. Eur J Hum Genet.

[42] Johnson KR, Erway LC, Cook SA, Willott JF, Zheng QY (1997). A major gene affecting age-related hearing loss in C57BL/6J mice. Hear Res.

[43] Johnson KR, Zheng QY, Erway LC (2000). A major gene affecting age-related hearing loss is common to at least ten inbred strains of mice. Genomics.

[44] Tajima S, Danzaki K, Ikeda K, Kamiya K (2020). Degradation and modification of cochlear gap junction proteins in the early development of age-related hearing loss. Exp Mol Med.

[45] Ingham NJ, Rook V, Di Domenico F, James E, Lewis MA, Girotto G, Buniello A, Steel KP (2020). Functional analysis of candidate genes from genome-wide association studies of hearing. Hear Res.

[46] Lewerenz J, Maher P (2015). Chronic glutamate toxicity in neurodegenerative diseases-what is the evidence?. Front Neurosci.

[47] Pujol R, Rebillard G, Puel JL, Lenoir M, Eybalin M, Recasens M (1990). Glutamate neurotoxicity in the cochlea: a possible consequence of ischaemic or anoxic conditions occurring in ageing. Acta Otolaryngol Suppl.

[48] Newman DL, Fisher LM, Ohmen J, Parody R, Fong CT, Frisina ST, Mapes F, Eddins DA, Robert Frisina D, Frisina RD, Friedman RA (2012). GRM7 variants associated with age-related hearing loss based on auditory perception. Hear Res.

[49] Friedman RA, Van Laer L, Huentelman MJ, Sheth SS, Van Eyken E, Corneveaux JJ, Tembe WD, Halperin RF, Thorburn AQ, Thys S, Bonneux S, Fransen E, Huyghe J, Pyykko I, Cremers CW, Kremer H, Dhooge I, Stephens D, Orzan E, Pfister M, Bille M, Parving A, Sorri M, Van de Heyning PH, Makmura L, Ohmen JD, Linthicum FH, Fayad JN, Pearson JV, Craig DW, Stephan DA, Van Camp G (2009). GRM7 variants confer susceptibility to age-related hearing impairment. Hum Mol Genet.

[50] Deng T, Li J, Liu J, Xu F, Liu X, Mi J, Bergquist J, Wang H, Yang C, Lu L, Song X, Yao C, Tian G, Zheng QY (2021). Hippocampal transcriptome-wide association study reveals correlations between impaired glutamatergic synapse pathway and age-related hearing loss in BXD-recombinant inbred mice. Front Neurosci.

[51] Unal M, Tamer L, Dogruer ZN, Yildirim H, Vayisoglu Y, Camdeviren H (2005). N-acetyltransferase 2 gene polymorphism and presbycusis. Laryngoscope.

[52] Bared A, Ouyang X, Angeli S, Du LL, Hoang K, Yan D, Liu XZ (2010). Antioxidant enzymes, presbycusis, and ethnic variability. Otolaryngol Head Neck Surg.

[53] Nolan LS, Cadge BA, Gomez-Dorado M, Dawson SJ (2013). A functional and genetic analysis of SOD2 promoter variants and their contribution to age-related hearing loss. Mech Ageing Dev.

[54] Arsenijevic D, Onuma H, Pecqueur C, Raimbault S, Manning BS, Miroux B, Couplan E, Alves-Guerra MC, Goubern M, Surwit R, Bouillaud F, Richard D, Collins S, Ricquier D (2000). Disruption of the uncoupling protein-2 gene in mice reveals a role in immunity and reactive oxygen species production. Nat Genet.

[55] Sugiura S, Uchida Y, Nakashima T, Ando F, Shimokata H (2010). The association between gene polymorphisms in uncoupling proteins and hearing impairment in Japanese elderly. Acta Otolaryngol.

[56] Bai U, Seidman MD, Hinojosa R, Quirk WS (1997). Mitochondrial DNA deletions associated with aging and possibly presbycusis: a human archival temporal bone study. Am J Otol.

[57] Fischel-Ghodsian N, Bykhovskaya Y, Taylor K, Kahen T, Cantor R, Ehrenman K, Smith R, Keithley E (1997). Temporal bone analysis of patients with presbycusis reveals high frequency of mitochondrial mutations. Hear Res.

[58] Miura S, Sasaki A, Kasai S, Sugawara T, Maeda Y, Goto S, Kasai T, Shimizume N, Jung S, Iwane T, Itoh K, Matsubara A (2022). Association of mitochondrial DNA haplogroup and hearing impairment with aging in Japanese general population of the Iwaki Health Promotion Project. J Hum Genet.

[59] Chen GD, Fechter LD (2003). The relationship between noise-induced hearing loss and hair cell loss in rats. Hear Res.

[60] Tarnowski BI, Schmiedt RA, Hellstrom LI, Lee FS, Adams JC (1991). Age-related changes in cochleas of mongolian gerbils. Hear Res.

[61] Fetoni AR, Pisani A, Rolesi R, Paciello F, Viziano A, Moleti A, Sisto R, Troiani D, Paludetti G, Grassi C (2022). Early noise-induced hearing loss accelerates presbycusis altering aging processes in the cochlea. Front Aging Neurosci.

[62] Masterson EA, Tak S, Themann CL, Wall DK, Groenewold MR, Deddens JA, Calvert GM (2013). Prevalence of hearing loss in the United States by industry. Am J Ind Med.

[63] Rosen S, Bergman M, Plester D, El-Mofty A, Satti MH (1962). Presbycusis study of a relatively noise-free population in the Sudan. Ann Otol Rhinol Laryngol.

[64] Goycoolea MV, Goycoolea HG, Farfan CR, Rodriguez LG, Martinez GC, Vidal R (1986). Effect of life in industrialized societies on hearing in natives of Easter Island. Laryngoscope.

[65] Spicer SS, Schulte BA (2002). Spiral ligament pathology in quiet-aged gerbils. Hear Res.

[66] Dawes P, Cruickshanks KJ, Moore DR, Edmondson-Jones M, McCormack A, Fortnum H, Munro KJ (2014). Cigarette smoking, passive smoking, alcohol consumption, and hearing loss. J Assoc Res Otolaryngol.

[67] Bae SH, Kwak SH, Choi JY, Jung J (2020). Synergistic effect of smoking on age-related hearing loss in patients with diabetes. Sci Rep.

[68] Ooi TC, Ishak WS, Sharif R, Shahar S, Rajab NF, Singh DKA, Mukari SZS (2021). Multidimensional risk factors of age-related hearing loss among malaysian community-dwelling older adults. Clin Interv Aging.

[69] Park DJ, Ha S, Choi JS, Lee SH, Park JE, Seo YJ (2020). Induced short-term hearing loss due to stimulation of age-related factors by intermittent hypoxia, high-fat diet, and galactose injection. Int J Mol Sci.

[70] Fransen E, Topsakal V, Hendrickx JJ, Van Laer L, Huyghe JR, Van Eyken E, Lemkens N, Hannula S, Maki-Torkko E, Jensen M, Demeester K, Tropitzsch A, Bonaconsa A, Mazzoli M, Espeso A, Verbruggen K, Huyghe J, Huygen PL, Kunst S, Manninen M, Diaz-Lacava A, Steffens M, Wienker TF, Pyykko I, Cremers CW, Kremer H, Dhooge I, Stephens D, Orzan E, Pfister M, Bille M, Parving A, Sorri M, Van de Heyning P, Van Camp G (2008). Occupational noise, smoking, and a high body mass index are risk factors for age-related hearing impairment and moderate alcohol consumption is protective: a European population-based multicenter study. J Assoc Res Otolaryngol.

[71] Puga AM, Pajares MA, Varela-Moreiras G, Partearroyo T (2018). Interplay between nutrition and hearing loss: state of art. Nutrients.

[72] Choi JE, Ahn J, Moon IJ (2021). Associations between age-related hearing loss and dietary assessment using data from Korean national health and nutrition examination survey. Nutrients.

[73] Kim TS, Chung JW (2019). Associations of dietary riboflavin, niacin, and retinol with age-related hearing loss: an analysis of Korean National health and nutrition examination survey data. Nutrients.

[74] Kang MJ, Lee Y, Kim YJ, Lee SY, Lee JG, Yi YH, Cho YH, Tak YJ, Park EJ, Lee SH, Kim GL, Choi JI, Ra YJ, Lee SR, Kwon RJ, Son SM, Lee YJ, Choi YE (2023). Association between sleep duration and presbycusis in Korean adults: Korea National Health and Nutrition Examination Survey. Korean J Fam Med.

[75] Hong JW, Jeon JH, Ku CR, Noh JH, Yoo HJ, Kim DJ (2015). The prevalence and factors associated with hearing impairment in the Korean adults: the 2010–2012 Korea National Health and Nutrition Examination Survey (observational study). Medicine (Baltimore).

[76] Tang D, Tran Y, Dawes P, Gopinath B (2023). A narrative review of lifestyle risk factors and the role of oxidative stress in age-related hearing loss. Antioxidants (Basel).

[77] Li Y, Womer RB, Silber JH (2004). Predicting cisplatin ototoxicity in children: the influence of age and the cumulative dose. Eur J Cancer.

[78] McKeage MJ (1995). Comparative adverse effect profiles of platinum drugs. Drug Saf.

[79] Ganesan P, Schmiedge J, Manchaiah V, Swapna S, Dhandayutham S, Kothandaraman PP (2018). Ototoxicity: a challenge in diagnosis and treatment. J Audiol Otol.

[80] Xie J, Talaska AE, Schacht J (2011). New developments in aminoglycoside therapy and ototoxicity. Hear Res.

[81] Yang CH, Schrepfer T, Schacht J (2015). Age-related hearing impairment and the triad of acquired hearing loss. Front Cell Neurosci.

[82] Beyea JA, Agrawal SK, Parnes LS (2012). Recent advances in viral inner ear disorders. Curr Opin Otolaryngol Head Neck Surg.

[83] Harris JP (1983). Immunology of the inner ear: response of the inner ear to antigen challenge. Otolaryngol Head Neck Surg.

[84] Okano T (2014). Immune system of the inner ear as a novel therapeutic target for sensorineural hearing loss. Front Pharmacol.

[85] Tornabene SV, Sato K, Pham L, Billings P, Keithley EM (2006). Immune cell recruitment following acoustic trauma. Hear Res.

[86] Menardo J, Tang Y, Ladrech S, Lenoir M, Casas F, Michel C, Bourien J, Ruel J, Rebillard G, Maurice T, Puel JL, Wang J (2012). Oxidative stress, inflammation, and autophagic stress as the key mechanisms of premature age-related hearing loss in SAMP8 mouse Cochlea. Antioxid Redox Signal.

[87] Peng L, Li N, Huang Z, Qiu C, Yin S (2022). Prognostic gene expression signature for age-related hearing loss. Front Med Lausanne.

[88] Corso JF (1963). Age and sex differences in pure-tone thresholds. Survey of hearing levels from 18 to 65 years. Arch Otolaryngol.

[89] Hultcrantz M, Simonoska R, Stenberg AE (2006). Estrogen and hearing: a summary of recent investigations. Acta Otolaryngol.

[90] Bonnard A, Bark R, Hederstierna C (2019). Clinical update on sensorineural hearing loss in Turner syndrome and the X-chromosome. Am J Med Genet C Semin Med Genet.

[91] Goman AM, Lin FR (2016). Prevalence of hearing loss by severity in the United States. Am J Public Health.

[92] Rousset F, Nacher-Soler G, Coelho M, Ilmjarv S, Kokje VBC, Marteyn A, Cambet Y, Perny M, Roccio M, Jaquet V, Senn P, Krause KH (2020). Redox activation of excitatory pathways in auditory neurons as mechanism of age-related hearing loss. Redox Biol.

[93] Islam MT (2017). Oxidative stress and mitochondrial dysfunction-linked neurodegenerative disorders. Neurol Res.

[94] Massaad CA, Klann E (2011). Reactive oxygen species in the regulation of synaptic plasticity and memory. Antioxid Redox Signal.

[95] Kamogashira T, Hayashi K, Fujimoto C, Iwasaki S, Yamasoba T (2017). Functionally and morphologically damaged mitochondria observed in auditory cells under senescence-inducing stress. NPJ Aging Mech Dis.

[96] Keithley EM, Canto C, Zheng QY, Wang X, Fischel-Ghodsian N, Johnson KR (2005). Cu/Zn superoxide dismutase and age-related hearing loss. Hear Res.

[97] Rahman K (2007). Studies on free radicals, antioxidants, and co-factors. Clin Interv Aging.

[98] Serra LSM, Araujo JG, Vieira ALS, Silva EMD, Andrade RR, Kuckelhaus SAS, Sampaio ALL (2020). Role of melatonin in prevention of age-related hearing loss. PLoS ONE.

[99] Jg DEA, Serra LSM, Lauand L, Kuckelhaus SAS, Sampaio ALL (2019). Protective effect of melatonin on cisplatin-induced ototoxicity in rats. Anticancer Res.

[100] Karlidag T, Yalcin S, Ozturk A, Ustundag B, Gok U, Kaygusuz I, Susaman N (2002). The role of free oxygen radicals in noise induced hearing loss: effects of melatonin and methylprednisolone. Auris Nasus Larynx.

[101] Serra LSM, Araujo JG, Novanta G, Lauand L, Silva EMD, Kuckelhaus SAS, Sampaio ALL (2022). Melatonin prevents age-related hearing loss in the murin experimental model. Braz J Otorhinolaryngol.

[102] Seidman MD, Khan MJ, Bai U, Shirwany N, Quirk WS (2000). Biologic activity of mitochondrial metabolites on aging and age-related hearing loss. Am J Otol.

[103] Kang JW, Choi HS, Kim K, Choi JY (2014). Dietary vitamin intake correlates with hearing thresholds in the older population: the Korean National Health and Nutrition Examination Survey. Am J Clin Nutr.

[104] Aladag I, Guven M, Songu M (2016). Prevention of gentamicin ototoxicity with N-acetylcysteine and vitamin A. J Laryngol Otol.

[105] Sha SH, Kanicki A, Halsey K, Wearne KA, Schacht J (2012). Antioxidant-enriched diet does not delay the progression of age-related hearing loss. Neurobiol Aging.

[106] Choo OS, Lee YY, Kim YS, Kim YJ, Lee DH, Kim H, Jang JH, Choung YH (2022). Effect of statin on age-related hearing loss via drug repurposing. Biochim Biophys Acta Mol Cell Res.

[107] Tan WJT, Song L (2023). Role of mitochondrial dysfunction and oxidative stress in sensorineural hearing loss. Hear Res.

[108] Bai X, Yao L, Ma X, Xu X (2018). Small molecules as SIRT modulators. Mini Rev Med Chem.

[109] Dai H, Sinclair DA, Ellis JL, Steegborn C (2018). Sirtuin activators and inhibitors: promises, achievements, and challenges. Pharmacol Ther.

[110] Fremont L (2000). Biological effects of resveratrol. Life Sci.

[111] Yumusakhuylu AC, Yazici M, Sari M, Binnetoglu A, Kosemihal E, Akdas F, Sirvanci S, Yuksel M, Uneri C, Tutkun A (2012). Protective role of resveratrol against cisplatin induced ototoxicity in guinea pigs. Int J Pediatr Otorhinolaryngol.

[112] Bonabi S, Caelers A, Monge A, Huber A, Bodmer D (2008). Resveratrol protects auditory hair cells from gentamicin toxicity. Ear Nose Throat J.

[113] Seidman M, Babu S, Tang W, Naem E, Quirk WS (2003). Effects of resveratrol on acoustic trauma. Otolaryngol Head Neck Surg.

[114] Muderris T, Yar Saglam AS, Unsal D, Mulazimoglu S, Sevil E, Kayhan H (2022). Efficiency of resveratrol in the prevention and treatment of age-related hearing loss. Exp Ther Med.

[115] Yang Z, Zhang Y, Yang S, Ding Y, Qu Y (2022). Low-dose resveratrol inhibits RIPK3-mediated necroptosis and delays the onset of age-related hearing loss. Front Pharmacol.

[116] Xiong H, Pang J, Yang H, Dai M, Liu Y, Ou Y, Huang Q, Chen S, Zhang Z, Xu Y, Lai L, Zheng Y (2015). Activation of miR-34a/SIRT1/p53 signaling contributes to cochlear hair cell apoptosis: implications for age-related hearing loss. Neurobiol Aging.

[117] Fang J, Wu H, Zhang J, Mao S, Shi H, Yu D, Chen Z, Su K, Xing Y, Dong H, Shi H (2022). A reduced form of nicotinamide riboside protects the cochlea against aminoglycoside-induced ototoxicity by SIRT1 activation. Biomed Pharmacother.

[118] Dinh CT, Goncalves S, Bas E, Van De Water TR, Zine A (2015). Molecular regulation of auditory hair cell death and approaches to protect sensory receptor cells and/or stimulate repair following acoustic trauma. Front Cell Neurosci.

[119] Fujioka M, Kanzaki S, Okano HJ, Masuda M, Ogawa K, Okano H (2006). Proinflammatory cytokines expression in noise-induced damaged cochlea. J Neurosci Res.

[120] Plontke SK, Meisner C, Agrawal S, Caye-Thomasen P, Galbraith K, Mikulec AA, Parnes L, Premakumar Y, Reiber J, Schilder AG, Liebau A (2022). Intratympanic corticosteroids for sudden sensorineural hearing loss. Cochrane Database Syst Rev.

[121] McCabe BF (2004). Autoimmune sensorineural hearing loss. 1979. Ann Otol Rhinol Laryngol.

[122] Ronchetti S, Migliorati G, Bruscoli S, Riccardi C (2018). Defining the role of glucocorticoids in inflammation. Clin Sci (Lond).

[123] Wakabayashi K, Fujioka M, Kanzaki S, Okano HJ, Shibata S, Yamashita D, Masuda M, Mihara M, Ohsugi Y, Ogawa K, Okano H (2010). Blockade of interleukin-6 signaling suppressed cochlear inflammatory response and improved hearing impairment in noise-damaged mice cochlea. Neurosci Res.

[124] Arpornchayanon W, Canis M, Ihler F, Settevendemie C, Strieth S (2013). TNF-alpha inhibition using etanercept prevents noise-induced hearing loss by improvement of cochlear blood flow in vivo. Int J Audiol.

[125] Dinarello CA, Simon A, van der Meer JW (2012). Treating inflammation by blocking interleukin-1 in a broad spectrum of diseases. Nat Rev Drug Discov.

[126] Iwai H, Inaba M, Van Bui D, Suzuki K, Sakagami T, Yun Y, Mitani A, Kobayashi Y, Kanda A (2021). Treg and IL-1 receptor type 2-expressing CD4(+) T cell-deleted CD4(+) T cell fraction prevents the progression of age-related hearing loss in a mouse model. J Neuroimmunol.

[127] Lamm K, Arnold W (2000). The effect of blood flow promoting drugs on cochlear blood flow, perilymphatic pO(2) and auditory function in the normal and noise-damaged hypoxic and ischemic guinea pig inner ear. Hear Res.

[128] Prazma J, Carrasco VN, Butler B, Waters G, Anderson T, Pillsbury HC (1990). Cochlear microcirculation in young and old gerbils. Arch Otolaryngol Head Neck Surg.

[129] Didier A, Miller JM, Nuttall AL (1993). The vascular component of sodium salicylate ototoxicity in the guinea pig. Hear Res.

[130] Alvarado JC, Fuentes-Santamaria V, Melgar-Rojas P, Valero ML, Gabaldon-Ull MC, Miller JM, Juiz JM (2015). Synergistic effects of free radical scavengers and cochlear vasodilators: a new otoprotective strategy for age-related hearing loss. Front Aging Neurosci.

[131] Han JS, Kim YL, Yu HJ, Park JM, Kim YJ, Choung YH, Park SY, Park SN (2022). Safety and efficacy of intratympanic histamine injection as an adjuvant to dexamethasone in a noise-induced murine model. Eur J Pharm Sci.

[132] Liu Y, Chu H, Chen J, Zhou L, Chen Q, Yu Y, Wu Z, Wang S, Lai Y, Pan C, Cui Y (2014). Age-related change in the expression of NKCC1 in the cochlear lateral wall of C57BL/6J mice. Acta Otolaryngol.

[133] Schulte BA, Schmiedt RA (1992). Lateral wall Na, K-ATPase and endocochlear potentials decline with age in quiet-reared gerbils. Hear Res.

[134] Chen J, Chu H, Xiong H, Yu Y, Huang X, Zhou L, Chen Q, Bing D, Liu Y, Wang S, Cui Y (2013). Downregulation of Cav1.3 calcium channel expression in the cochlea is associated with age-related hearing loss in C57BL/6J mice. NeuroReport.

[135] Qi F, Zhang R, Chen J, Zhao F, Sun Y, Du Z, Bing D, Li P, Shao S, Zhu H, Chu H (2019). Down-regulation of Cav13 in auditory pathway promotes age-related hearing loss by enhancing calcium-mediated oxidative stress in male mice. Aging (Albany NY).

[136] Leitner MG, Halaszovich CR, Oliver D (2011). Aminoglycosides inhibit KCNQ4 channels in cochlear outer hair cells via depletion of phosphatidylinositol(4,5)bisphosphate. Mol Pharmacol.

[137] Sheppard AM, Chen GD, Salvi R (2015). Potassium ion channel openers, Maxipost and Retigabine, protect against peripheral salicylate ototoxicity in rats. Hear Res.

[138] Lei D, Gao X, Perez P, Ohlemiller KK, Chen CC, Campbell KP, Hood AY, Bao J (2011). Anti-epileptic drugs delay age-related loss of spiral ganglion neurons via T-type calcium channel. Hear Res.

[139] Yu YF, Wu WY, Xiao GS, Shi J, Ling HY (2015). Effect of T-type calcium channel blockers on spiral ganglion neurons of aged C57BL/6J mice. Int J Clin Exp Med.

[140] Yu YF, Wu WY, Xiao GS, Ling HY, Pan C (2016). Protection of the cochlear hair cells in adult C57BL/6J mice by T-type calcium channel blockers. Exp Ther Med.

[141] Hetz CA, Torres V, Quest AF (2005). Beyond apoptosis: nonapoptotic cell death in physiology and disease. Biochem Cell Biol.

[142] Kim R, Emi M, Tanabe K (2006). Role of mitochondria as the gardens of cell death. Cancer Chemother Pharmacol.

[143] Dhanasekaran DN, Reddy EP (2008). JNK signaling in apoptosis. Oncogene.

[144] Cheng AG, Cunningham LL, Rubel EW (2005). Mechanisms of hair cell death and protection. Curr Opin Otolaryngol Head Neck Surg.

[145] Sha SH, Chen FQ, Schacht J (2009). Activation of cell death pathways in the inner ear of the aging CBA/J mouse. Hear Res.

[146] Sugahara K, Rubel EW, Cunningham LL (2006). JNK signaling in neomycin-induced vestibular hair cell death. Hear Res.

[147] Wang J, Van De Water TR, Bonny C, de Ribaupierre F, Puel JL, Zine A (2003). A peptide inhibitor of c-Jun N-terminal kinase protects against both aminoglycoside and acoustic trauma-induced auditory hair cell death and hearing loss. J Neurosci.

[148] Battaglia A, Pak K, Brors D, Bodmer D, Frangos JA, Ryan AF (2003). Involvement of ras activation in toxic hair cell damage of the mammalian cochlea. Neuroscience.

[149] Salvinelli F, Casale M, Greco F, Trivelli M, Di Peco V, Amendola T, Antonelli A, Stampachiacchiere B, Aloe L (2002). Nerve growth factor serum level is reduced in patients with sensorineural hearing impairment: possible clinical implications. J Biol Regul Homeost Agents.

[150] Celaya AM, Rodriguez-de la Rosa L, Bermudez-Munoz JM, Zubeldia JM, Roma-Mateo C, Avendano C, Pallardo FV, Varela-Nieto I (2021). IGF-1 haploinsufficiency causes age-related chronic cochlear inflammation and increases noise-induced hearing loss. Cells.

[151] Gao L, Ge R, Xie G, Yao D, Li P, Wang O, Ma X, Han F (2017). Hearing Improvement in A/J Mice via the mouse nerve growth factor. Clin Exp Otorhinolaryngol.

[152] Szobota S, Mathur PD, Siegel S, Black K, Saragovi HU, Foster AC (2019). BDNF, NT-3 and Trk receptor agonist monoclonal antibodies promote neuron survival, neurite extension, and synapse restoration in rat cochlea ex vivo models relevant for hidden hearing loss. PLoS ONE.

[153] Liang Z, Gao M, Jia H, Han W, Zheng Y, Zhao Y, Yang H (2023). Analysis of clinical efficacy and influencing factors of nerve growth factor (NGF) treatment for sudden sensorineural hearing loss. Ear Nose Throat J.

[154] Hayashi Y, Yamamoto N, Nakagawa T, Ito J (2013). Insulin-like growth factor 1 inhibits hair cell apoptosis and promotes the cell cycle of supporting cells by activating different downstream cascades after pharmacological hair cell injury in neonatal mice. Mol Cell Neurosci.

[155] Glick HA, Sharma A (2020). Cortical neuroplasticity and cognitive function in early-stage, mild-moderate hearing loss: evidence of neurocognitive benefit from hearing aid use. Front Neurosci.

[156] Gurgel RK, Duff K, Foster NL, Urano KA, de Torres A (2022). Evaluating the impact of cochlear implantation on cognitive function in older adults. Laryngoscope.

[157] Linthicum FH, Fayad J, Otto SR, Galey FR, House WF (1991). Cochlear implant histopathology. Am J Otol.

[158] Moore BCJ (2022). Listening to music through hearing aids: potential lessons for cochlear implants. Trends Hear.

[159] Johnson CE, Jilla AM, Danhauer JL, Sullivan JC, Sanchez KR (2018). Benefits from, satisfaction with, and self-efficacy for advanced digital hearing aids in users with mild sensorineural hearing loss. Semin Hear.

[160] Ylikoski J, Mrena R, Makitie A, Kuokkanen J, Pirvola U, Savolainen S (2008). Hyperbaric oxygen therapy seems to enhance recovery from acute acoustic trauma. Acta Otolaryngol.

[161] Feng T, Zhang Q, Wei J, Wang X, Geng Y (2022). Effects of alprostadil combined with hyperbaric oxygen on hearing recovery and hemorheology in patients with sudden sensorineural hearing loss and analysis of related influencing factors. Exp Ther Med.

[162] Kuhn M, Heman-Ackah SE, Shaikh JA, Roehm PC (2011). Sudden sensorineural hearing loss: a review of diagnosis, treatment, and prognosis. Trends Amplif.

[163] Bazzi C, Venturini CT, Pagani C, Arrigo G, D'Amico G (1995). Hearing loss in short- and long-term haemodialysed patients. Nephrol Dial Transplant.

[164] Thodi C, Thodis E, Danielides V, Pasadakis P, Vargemezis V (2006). Hearing in renal failure. Nephrol Dial Transplant.

[165] Ling S, Xu JW (2016). Biological activities of 2,3,5,4'-tetrahydroxystilbene-2-O-beta-D-glucoside in antiaging and antiaging-related disease treatments. Oxid Med Cell Longev.

[166] Wu TY, Lin JN, Luo ZY, Hsu CJ, Wang JS, Wu HP (2020). 2,3,4',5-Tetrahydroxystilbene-2-O-beta-D-Glucoside (THSG) activates the Nrf2 antioxidant pathway and attenuates oxidative stress-induced cell death in mouse cochlear UB/OC-2 Cells. Biomolecules.

[167] Wen YH, Lin HY, Lin JN, Tseng GF, Hwang CF, Lin CC, Hsu CJ, Wu HP (2022). 2,3,4',5-Tetrahydroxystilbene-2-O-beta-D-glucoside ameliorates gentamicin-induced ototoxicity by modulating autophagy via Sesn2/AMPK/mTOR signaling. Int J Mol Med.

[168] Lin WS, Song XZ (1989). Clinical and experimental research on a kidney-tonifying prescription in preventing and treating children's hearing-loss induced by aminoglycoside antibiotic ototoxicity. Zhong Xi Yi Jie He Za Zhi.

[169] Wang Z (1989). Experimental study of Rhizoma drynariae (Gusuibu) in the treatment of streptomycin ototoxicity. Zhonghua Er Bi Yan Hou Ke Za Zhi.

[170] Long M, Smouha EE, Qiu D, Li F, Johnson F, Luft B (2004). Flavanoid of Drynaria fortunei protects against gentamicin ototoxicity. Phytother Res.

[171] Liu Q, Li N, Yang Y, Yan X, Dong Y, Peng Y, Shi J (2021). Prediction of the molecular mechanisms underlying erlong Zuoci treatment of age-related hearing loss via network pharmacology-based analyses combined with experimental validation. Front Pharmacol.

[172] Dong Y, Cao BY, Wang J, Ding DL, Han ZF, Shi JR (2010). Effects of Erlong Zuoci pill and its disassembled prescriptions on gentamicin-induced ototoxicity model in vitro. Chin J Integr Med.

[173] Yu HH, Hur JM, Seo SJ, Moon HD, Kim HJ, Park RK, You YO (2009). Protective effect of ursolic acid from Cornus officinalis on the hydrogen peroxide-induced damage of HEI-OC1 auditory cells. Am J Chin Med.

[174] Zhuang J, Zhang M, Zeng Z, Xu F, Han T, Hu S, Sun Y (1992). The use of 6-flavor Rehmannia decoction with additives in the prevention of ototoxic deafness induced by gentamicin in guinea pigs. Zhongguo Zhong Yao Za Zhi.

[175] Yu HH, Kim YH, Jung SY, Shin MK, Park RK, So HS, Kim KY, Lee DH, You YO (2006). Rehmannia glutinosa activates intracellular antioxidant enzyme systems in mouse auditory cells. Am J Chin Med.

[176] Yu HH, Seo SJ, Kim YH, Lee HY, Park RK, So HS, Jang SL, You YO (2006). Protective effect of Rehmannia glutinosa on the cisplatin-induced damage of HEI-OC1 auditory cells through scavenging free radicals. J Ethnopharmacol.

[177] Kim YH, Kim EY, Rodriguez I, Nam YH, Jeong SY, Hong BN, Choung SY, Kang TH (2020). *Sesamum*
*indicum* L. oil and sesamin induce auditory-protective effects through changes in hearing loss-related gene expression. J Med Food.

[178] Francis SP, Kramarenko II, Brandon CS, Lee FS, Baker TG, Cunningham LL (2011). Celastrol inhibits aminoglycoside-induced ototoxicity via heat shock protein 32. Cell Death Dis.

[179] Han Z, Gu YY, Cong N, Ma R, Chi FL (2018). Celastrol enhances Atoh1 expression in inner ear stem cells and promotes their differentiation into functional auditory neuronal-like cells. Organogenesis.

[180] Goldbach-Mansky R, Wilson M, Fleischmann R, Olsen N, Silverfield J, Kempf P, Kivitz A, Sherrer Y, Pucino F, Csako G, Costello R, Pham TH, Snyder C, van der Heijde D, Tao X, Wesley R, Lipsky PE (2009). Comparison of *Tripterygium*
*wilfordii* Hook F versus sulfasalazine in the treatment of rheumatoid arthritis: a randomized trial. Ann Intern Med.

[181] Liu YC, Liu GY, Liu RL (1995). Effects of poria cocos on ototoxicity induced by kanamycin in guinea-pigs. Zhongguo Zhong Xi Yi Jie He Za Zhi.

[182] Yin LM, Zhang GQ, Yan XK, Wang Y, Xu YD, Yang YQ (2013). An in vivo and in vitro evaluation of the mutual interactions between the lung and the large intestine. Evid Based Complement Alternat Med.

[183] Zhang Z, Lam TN, Zuo Z (2013). Radix Puerariae: an overview of its chemistry, pharmacology, pharmacokinetics, and clinical use. J Clin Pharmacol.

[184] Chen W, Yao Q, Liu W, Zhang B, Wang Y, Liu B (2009). Preventive effects of pueraria on presbycusis in rats. Lin Chung Er Bi Yan Hou Tou Jing Wai Ke Za Zhi.

[185] Qu J, Liao YH, Kou ZZ, Wei YY, Huang J, Chen J, Yanagawa Y, Wu SX, Shi M, Li YQ (2015). Puerarin alleviates noise-induced hearing loss via affecting PKCgamma and GABAB receptor expression. J Neurol Sci.

[186] Xiong M, He Q, Lai H, Huang W, Wang L, Yang C (2012). Radix astragali injection enhances recovery from sudden deafness. Am J Otolaryngol.

[187] Xiong M, Zhu Y, Lai H, Fu X, Deng W, Yang C, He Q, Zheng G (2015). Radix astragali inhibits the down-regulation of connexin 26 in the stria vascularis of the guinea pig cochlea after acoustic trauma. Eur Arch Otorhinolaryngol.

[188] Xiong M, He Q, Lai H, Wang J (2012). Astragaloside IV inhibits apoptotic cell death in the guinea pig cochlea exposed to impulse noise. Acta Otolaryngol.

[189] Chen Q, Rahman K, Wang SJ, Zhou S, Zhang H (2020). *Scutellaria*
*barbata*: a review on chemical constituents, pharmacological activities and clinical applications. Curr Pharm Des.

[190] Kang TH, Hong BN, Park C, Kim SY, Park R (2010). Effect of baicalein from *Scutellaria*
*baicalensis* on prevention of noise-induced hearing loss. Neurosci Lett.

[191] Rodriguez I, Hong BN, Nam YH, Kim EY, Park GH, Ji MG, Kang TH (2017). Bioconversion of *Scutellaria*
*baicalensis* extract can increase recovery of auditory function in a mouse model of noise-induced hearing loss. Biomed Pharmacother.

[192] Zhang X, Yu J (2019). Baicalin attenuates gentamicin-induced cochlear hair cell ototoxicity. J Appl Toxicol.

[193] Olgun Y, Kirkim G, Altun Z, Aktas S, Kolatan E, Kiray M, Bagriyanik A, Olgun A, Cakir Kizmazoglu D, Ozogul C, Ellidokuz H, Ercetin P, Serbetcioglu B, Yilmaz O, Guneri EA (2016). Protective effect of Korean red ginseng on cisplatin ototoxicity: is it effective enough?. J Int Adv Otol.

[194] Tian C, Kim YH, Kim YC, Park KT, Kim SW, Kim YJ, Lim HJ, Choung YH (2013). Korean red ginseng ameliorates acute 3-nitropropionic acid-induced cochlear damage in mice. Neurotoxicology.

[195] Tian CJ, Kim SW, Kim YJ, Lim HJ, Park R, So HS, Choung YH (2013). Red ginseng protects against gentamicin-induced balance dysfunction and hearing loss in rats through antiapoptotic functions of ginsenoside Rb1. Food Chem Toxicol.

[196] Hong BN, Kim SY, Yi TH, Kang TH (2011). Post-exposure treatment with ginsenoside compound K ameliorates auditory functional injury associated with noise-induced hearing loss in mice. Neurosci Lett.

[197] Tian C, Kim YJ, Lim HJ, Kim YS, Park HY, Choung YH (2014). Red ginseng delays age-related hearing and vestibular dysfunction in C57BL/6 mice. Exp Gerontol.

[198] Kim TS, Lee HS, Chung JW (2015). The effect of Korean Red Ginseng on symptoms and quality of life in chronic tinnitus: a randomized. Open-Label Pilot Study J Audiol Otol.

[199] Doosti A, Lotfi Y, Moossavi A, Bakhshi E, Talasaz AH, Hoorzad A (2014). Comparison of the effects of N-acetyl-cysteine and ginseng in prevention of noise induced hearing loss in male textile workers. Noise Health.

[200] Yu Y, Hu B, Bao J, Mulvany J, Bielefeld E, Harrison RT, Neton SA, Thirumala P, Chen Y, Lei D, Qiu Z, Zheng Q, Ren J, Perez-Flores MC, Yamoah EN, Salehi P (2018). Otoprotective effects of *Stephania*
*tetrandra* S. moore herb isolate against acoustic trauma. J Assoc Res Otolaryngol.

[201] Uzun L, Balbaloglu E, Akinci H (2012). Garlic-supplemented diet attenuates gentamicin-induced ototoxicity: an experimental study. Ann Otol Rhinol Laryngol.

[202] Wu X, Li X, Song Y, Li H, Bai X, Liu W, Han Y, Xu L, Li J, Zhang D, Wang H, Fan Z (2017). Allicin protects auditory hair cells and spiral ganglion neurons from cisplatin - Induced apoptosis. Neuropharmacology.

[203] Uzun L, Kokten N, Cam OH, Kalcioglu MT, Ugur MB, Tekin M, Acar GO (2016). The effect of garlic derivatives (S-Allylmercaptocysteine, Diallyl Disulfide, and S-Allylcysteine) on gentamicin induced ototoxicity: an experimental study. Clin Exp Otorhinolaryngol.

[204] Stange G, Benning CD (1975). The influence on sound damages by an extract of ginkgo biloba (author's transl). Arch Otorhinolaryngol.

[205] Tziridis K, Korn S, Ahlf S, Schulze H (2014). Protective effects of Ginkgo biloba extract EGb 761 against noise trauma-induced hearing loss and tinnitus development. Neural Plast.

[206] Abdel-Kader R, Hauptmann S, Keil U, Scherping I, Leuner K, Eckert A, Muller WE (2007). Stabilization of mitochondrial function by Ginkgo biloba extract (EGb 761). Pharmacol Res.

[207] Eckert A, Keil U, Scherping I, Hauptmann S, Muller WE (2005). Stabilization of mitochondrial membrane potential and improvement of neuronal energy metabolism by Ginkgo biloba extract EGb 761. Ann N Y Acad Sci.

[208] Luo Y, Smith JV, Paramasivam V, Burdick A, Curry KJ, Buford JP, Khan I, Netzer WJ, Xu H, Butko P (2002). Inhibition of amyloid-beta aggregation and caspase-3 activation by the Ginkgo biloba extract EGb761. Proc Natl Acad Sci U S A.

[209] Schindowski K, Leutner S, Kressmann S, Eckert A, Muller WE (2001). Age-related increase of oxidative stress-induced apoptosis in mice prevention by Ginkgo biloba extract (EGb761). J Neural Transm (Vienna).

[210] Yang TH, Young YH, Liu SH (2011). EGb 761 (Ginkgo biloba) protects cochlear hair cells against ototoxicity induced by gentamicin via reducing reactive oxygen species and nitric oxide-related apoptosis. J Nutr Biochem.

[211] Wang C, Han Z (2015). Ginkgo Biloba extract enhances differentiation and performance of neural stem cells in mouse cochlea. Cell Mol Neurobiol.

[212] Dogan R, Sjostrand AP, Yenigun A, Karatas E, Kocyigit A, Ozturan O (2018). Influence of Ginkgo Biloba extract (EGb 761) on expression of IL-1 Beta, IL-6, TNF-alfa, HSP-70, HSF-1 and COX-2 after noise exposure in the rat cochlea. Auris Nasus Larynx.

[213] Tian CJ, Kim YJ, Kim SW, Lim HJ, Kim YS, Choung YH (2013). A combination of cilostazol and Ginkgo biloba extract protects against cisplatin-induced Cochleo-vestibular dysfunction by inhibiting the mitochondrial apoptotic and ERK pathways. Cell Death Dis.

[214] Chang MY, Rhee J, Kim SH, Kim YH (2018). The protective effect of Egb 761 against 3-Nitropropionic acid-induced hearing loss: the role of Sirtuin 1. Clin Exp Otorhinolaryngol.

[215] Fukaya H, Kanno H (1999). Experimental studies of the protective effect of ginkgo biloba extract (GBE) on cisplatin-induced toxicity in rats. Nihon Jibiinkoka Gakkai Kaiho.

[216] Ma W, Hu J, Cheng Y, Wang J, Zhang X, Xu M (2015). Ginkgolide B protects against cisplatin-induced ototoxicity: enhancement of Akt-Nrf2-HO-1 signaling and reduction of NADPH oxidase. Cancer Chemother Pharmacol.

[217] Sun Y, Xing Y, Jiang X, Tao D, Hu L, Wang Y, Dong H (2021). Effectiveness of Ginkgo biloba diterpene lactone in the treatment of sudden sensorineural hearing loss. Am J Otolaryngol.

[218] Si X, Yu Z, Ren X, Huang L, Feng Y (2022). Efficacy and safety of standardized Ginkgo biloba L. leaves extract as an adjuvant therapy for sudden sensorineural hearing loss: a systematic review and meta-analysis. J Ethnopharmacol.

[219] Koo JW, Chang MY, Yun SC, Kim TS, Kong SK, Chung JW, Goh EK (2016). The efficacy and safety of systemic injection of Ginkgo biloba extract, EGb761, in idiopathic sudden sensorineural hearing loss: a randomized placebo-controlled clinical trial. Eur Arch Otorhinolaryngol.

[220] Kahya V, Ozucer B, Dogan R, Meric A, Yuksel M, Gedikli O, Ozturan O (2014). Pomegranate extract: a potential protector against aminoglycoside ototoxicity. J Laryngol Otol.

[221] Liu S, Xu T, Wu X, Lin Y, Bao D, Di Y, Ma T, Dang Y, Jia P, Xian J, Wang A, Liu Y (2017). Pomegranate peel extract attenuates D-galactose-induced oxidative stress and hearing loss by regulating PNUTS/PP1 activity in the mouse cochlea. Neurobiol Aging.

[222] Tanigawa T, Shibata R, Kondo K, Katahira N, Kambara T, Inoue Y, Nonoyama H, Horibe Y, Ueda H, Murohara T (2015). Soybean beta-conglycinin prevents age-related hearing impairment. PLoS ONE.

[223] Seidman MD, Khan MJ, Tang WX, Quirk WS (2002). Influence of lecithin on mitochondrial DNA and age-related hearing loss. Otolaryngol Head Neck Surg.

[224] Shi L, An Y, Wang A, Gao Q, Yang Y (2014). The protective effect of Salvia miltiorrhiza on gentamicin-induced ototoxicity. Am J Otolaryngol.

[225] Xu O, Liu Y, Li X, Yang Y, Zhang Z, Wang N, Zhang Y, Lu H (2011). Protective effects of Salvia miltiorrhiza against cisplatin-induced ototoxicity in guinea pigs. Am J Otolaryngol.

[226] Wang AM, Sha SH, Lesniak W, Schacht J (2003). Tanshinone (Salviae miltiorrhizae extract) preparations attenuate aminoglycoside-induced free radical formation in vitro and ototoxicity in vivo. Antimicrob Agents Chemother.

[227] Fetoni AR, Eramo SL, Paciello F, Rolesi R, Podda MV, Troiani D, Paludetti G (2014). Curcuma longa (curcumin) decreases in vivo cisplatin-induced ototoxicity through heme oxygenase-1 induction. Otol Neurotol.

[228] Fetoni AR, Paciello F, Mezzogori D, Rolesi R, Eramo SL, Paludetti G, Troiani D (2015). Molecular targets for anticancer redox chemotherapy and cisplatin-induced ototoxicity: the role of curcumin on pSTAT3 and Nrf-2 signalling. Br J Cancer.

[229] Monroe JD, Millay MH, Patty BG, Smith ME (2018). The curcuminoid, EF-24, reduces cisplatin-mediated reactive oxygen species in zebrafish inner ear auditory and vestibular tissues. J Clin Neurosci.

[230] Guthrie OW, Gearhart CA, Fulton S, Fechter LD (2011). Carboxy alkyl esters of *Uncaria*
*tomentosa* augment recovery of sensorineural functions following noise injury. Brain Res.

[231] Tang YT, Wu J, Bao MF, Tan QG, Cai XH (2022). Dimeric Erythrina alkaloids as well as their key units from Erythrina variegata. Phytochemistry.

[232] Tang YT, Wu J, Yu Y, Bao MF, Tan QG, Schinnerl J, Cai XH (2021). Colored dimeric alkaloids from the barks of *Erythrina*
*variegata* and their neuroprotective effects. J Org Chem.

[233] Karawani H, Bitan T, Attias J, Banai K (2015). Auditory perceptual learning in adults with and without age-related hearing loss. Front Psychol.

[234] Hu Z, Ulfendahl M (2006). Cell replacement therapy in the inner ear. Stem Cells Dev.

[235] Schweinfurth J, Pribitkin E (2003). Sudden hearing loss associated with ephedra use. Am J Health Syst Pharm.

[236] Chan YH, Liu TC, Liao CK, Cheng YF, Tsai CH, Lu YC, Hu CJ, Lin HJ, Lee YL, Wu CC, Hsu CJ (2019). Consumption of betel quid contributes to sensorineural hearing impairment through arecoline-induced oxidative stress. Sci Rep.

[237] Chuu JJ, Hsu CJ, Lin-Shiau SY (2001). Abnormal auditory brainstem responses for mice treated with mercurial compounds: involvement of excessive nitric oxide. Toxicology.

[238] Zhang XC, Xu XP, Xu WT, Hou WZ, Cheng YY, Li CX, Ni GX (2015). Acupuncture therapy for sudden sensorineural hearing loss: a systematic review and meta-analysis of randomized controlled trials. PLoS ONE.

